# Nanomaterials for Healthcare Biosensing Applications

**DOI:** 10.3390/s19235311

**Published:** 2019-12-02

**Authors:** Muqsit Pirzada, Zeynep Altintas

**Affiliations:** Technical University of Berlin, Straße des 17. Juni 124, 10623 Berlin, Germany; muqsit.pirzada@campus.tu-berlin.de

**Keywords:** nanomaterials, carbon allotrope-based nanomaterials, inorganic nanomaterials, organic nanomaterials, healthcare biosensors, molecular machines

## Abstract

In recent years, an increasing number of nanomaterials have been explored for their applications in biomedical diagnostics, making their applications in healthcare biosensing a rapidly evolving field. Nanomaterials introduce versatility to the sensing platforms and may even allow mobility between different detection mechanisms. The prospect of a combination of different nanomaterials allows an exploitation of their synergistic additive and novel properties for sensor development. This paper covers more than 290 research works since 2015, elaborating the diverse roles played by various nanomaterials in the biosensing field. Hence, we provide a comprehensive review of the healthcare sensing applications of nanomaterials, covering carbon allotrope-based, inorganic, and organic nanomaterials. These sensing systems are able to detect a wide variety of clinically relevant molecules, like nucleic acids, viruses, bacteria, cancer antigens, pharmaceuticals and narcotic drugs, toxins, contaminants, as well as entire cells in various sensing media, ranging from buffers to more complex environments such as urine, blood or sputum. Thus, the latest advancements reviewed in this paper hold tremendous potential for the application of nanomaterials in the early screening of diseases and point-of-care testing.

## 1. Introduction

The International Union of Pure and Applied Chemistry (IUPAC) defines a biosensor as, “a device that uses specific biochemical reactions mediated by isolated enzymes, immunosystems, tissues, organelles or whole cells to detect chemical compounds, usually by electrical, thermal or optical signals” [1]. This definition facilitates an insight into what a biosensor entails. Biosensors have three imperative constituents [2]:

1. A receptor that specifically binds to an analyte;
2. A transducer that generates a signal following the binding event;
3. A detection system to quantify the signal and transform it into utile information.

These detection methods may be electrochemical, optical, or piezoelectric in nature. In contrast to conventional screening techniques, such as enzyme linked immunosorbent assays (ELISA), biosensors can be fully automated, show enhanced reproducibility, allow real-time and rapid analysis, and often show a possibility for re-use as a result of surface regeneration [3]. Biosensing plays a key role in a multitude of fields, such as medical diagnostics [2,4], food toxicity [5], fermentations [6], environmental safety [7], biodefense [8], and plant biology [9].

Ischaemic heart disease, lung cancer, cirrhosis, and similar infectious diseases are the leading causes of death worldwide [10]. Successful and inexpensive remedies are impeded by a lack of early diagnosis. Biosensors have thus gained prominence in the field of healthcare diagnostics by providing user-friendly, economical, reliable, and rapid sensing platforms [2]. Biosensing technology has considerable merits in comparison to conventional detection techniques involving spectroscopy or chromatography. These include an elimination of the need for skilled operating personnel, quicker response times, portability, and higher sensitivity [3]. For instance, the required detection time of pathogens such as anthrax has reduced from 2–3 days to 5 min with the help of modern biosensors [11].

**Figure 1:**
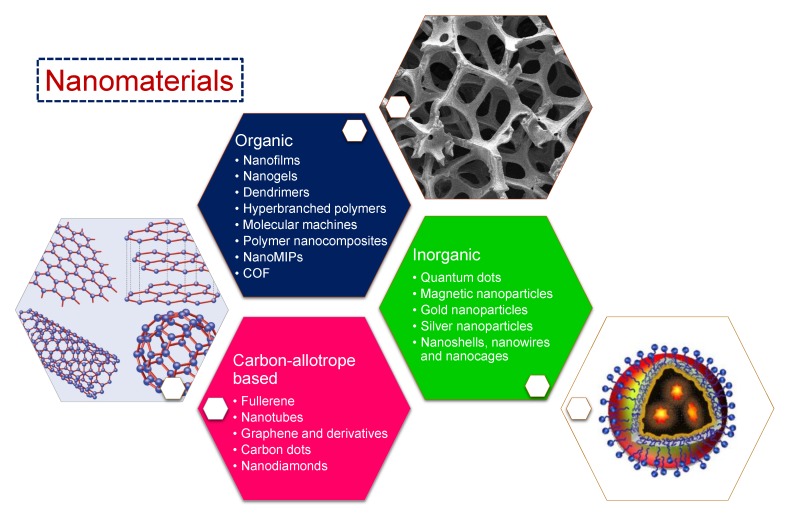
Various kinds of nanomaterials discussed in this review.

Materials with at least one of their dimensions measuring 1–100 nm are termed nanomaterials [12]. Due to their small size, most of their constituent atoms or molecules are located on the surface of such materials, giving rise to remarkable distinction in their fundamental physicochemical properties from the bulk of the same materials. Another factor causing significant differences in the characteristics of nanomaterials is the quantum effects arising from discontinuous behaviour because of the quantum confinement of delocalised electrons. Since the number of atoms on the surface of these nanoparticles is much higher than the bulk, they show less binding energy, thus exhibiting a lower melting point. The shape of these particles is crucial to their properties. For instance, nanorods may have significantly different properties to nanospheres of the same material [2]. The increased surface area per unit mass also results in an approximately 1000-fold increase in the chemical reactivity [13]. Synthetic nanostructures such as quantum dots rely on the exploitation of the quantum effects observed in nanoparticles. They act as artificial atoms, since their electronic behaviour is very similar to that of small molecules or individual atoms, as the spatial confinement of electrons at nanoscale generates a quantised energy spectrum. Similarly, owing to multiple unpaired electron spins from hundreds of atoms, nanoparticles possess magnetic moments, showing their best performance at 10–29 nm sizes because of supermagnetism, and are therefore suitable as contrast agents in magnetic resonance imaging (MRI) [12,13,14,15]. Due to all these factors, there are various possible classifications of nanomaterials. On the basis of chemical constitution, nanomaterials can mainly be classified into: (1) carbon allotrope-based nanomaterials consisting of only carbon atoms, (2) inorganic nanomaterials made up of metallic or non-metallic constituents such as Au, Ag, SiO_2_, and (3) organic nanomaterials majorly comprising of polymeric nanomaterials. Based on structural differences, each of these nanomaterials can be further categorised into several subtypes, as shown in Figure 1.

Nanomaterials can be engineered by following two main approaches—top-down and bottom-up approaches. In the top-down approach, a macroscale machine is designed and controlled to fabricate an exact replica of itself, but smaller in dimension. This smaller machine in turn produces an even smaller replica and the process is repeated until nanoscale dimensions are achieved. In the bottom-up approach, larger structures are built by the assembly of individual atoms or molecules with the help of biotechnology, scanning probes, or supramolecular chemistry [16]. Although both the aforementioned approaches play a vital role in the synthesis of nanomaterial-based biosensors, the bottom-up approach finds greater application.

**Figure 2:**
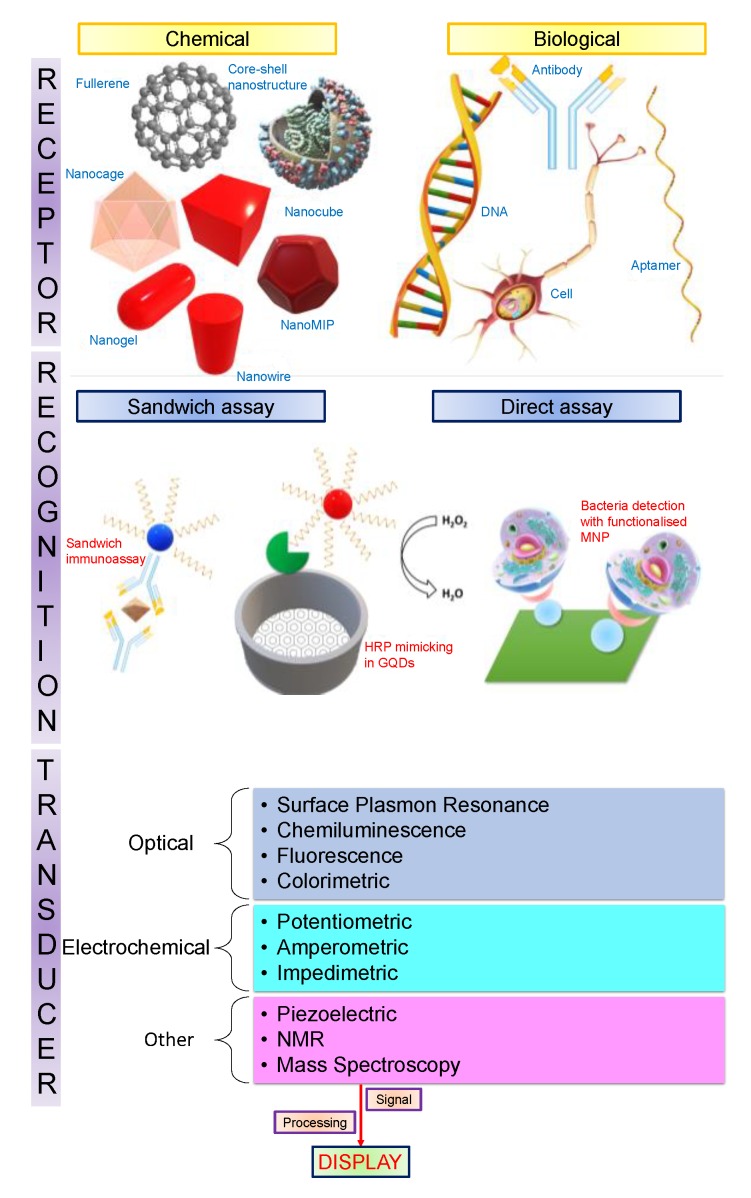
Roles of different nanomaterials in biosensing.

Nanotechnological advancements have spurred the development of assays and devices for medical diagnostics which are faster, cheaper, more sensitive, and more accurate. Biosensors utilising nanomaterials bring various disciplines together, such as chemistry, molecular engineering, material science, and biotechnology [3]. They offer extremely high sensitivity, such that some biosensors are now capable of detecting as low as one parasite per microlitre of blood [17]. The recognition of disease biomarkers at extremely low abundance, with the help of nanotechnology, permits the screening of diseases at very early stages. This has the capacity to upgrade medical techniques required for follow-up procedures and routine prognosis to monitor patient diagnosis. Furthermore, blending nanotechnology with biosensing is vital for point-of-care (POC) diagnosis in countries which lack advanced medical facilities [2]. Figure 2 presents a schematic of inorganic nanoparticles in association with various kinds of biomarkers and a linear flow representing each stage of biosensing from analyte recognition through transduction, and ultimately a measurable signal that is processed and displayed. In the following sections of this paper, we provide a comprehensive review on the application of nanomaterials in healthcare sensing, covering carbon allotrope-based nanomaterials, inorganic nanomaterials, and organic nanomaterials.

## 2. Carbon Allotrope-Based Nanomaterial Applications in Healthcare Biosensing

**Figure 3:**
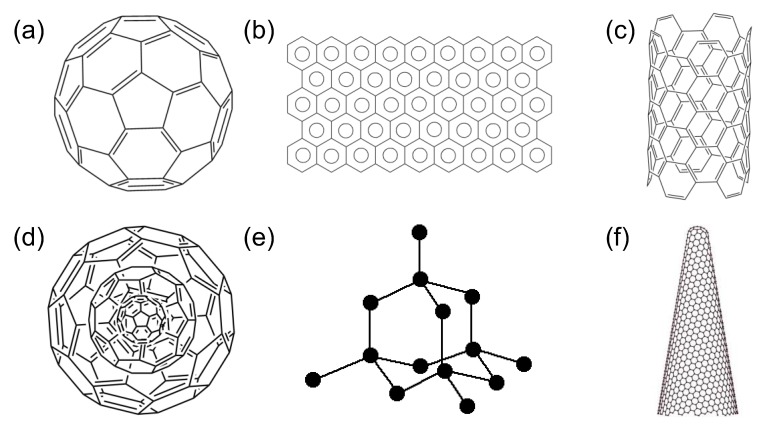
Various crystalline allotropes of carbon. (**a**) Fullerene—C^60^, (**b**) Graphene monolayer, (**c**) carbon nanotube (CNT), (**d**) carbon nanoonion, (**e**) nanodiamond, (**f**) carbon nanohorn.

Carbon allotrope-based nanomaterials have drawn plenty of research interest in the field of medical biosensing in the last few years. Because of the presence of a variety of carbon allotropes, such as graphite, fullerenes, diamonds and lonsdaleite, as well as more novel forms such as graphene, nanohorns, and nanotubes, as shown in Figure 3, carbon allotrope-based nanomaterials are highly valued [18]. Each of these allotropes possesses inimitable and unique features, which lead to their extensive exploitation for diverse biological applications, ranging from cancer therapy, tissue engineering, and drug delivery to medical diagnostics, bioimaging, and biosensing [19,20]. Carbon allotrope-based nanomaterials possess an unparalleled combination of optical, electrical, and mechanical properties, generating miniaturised sensors with superior performance and low power requirements. These nanomaterials are flexible and thermally stable in nature, with superior strength-to-weight ratio as well as high electron mobilities [21]. A broad spectrum of compounds that find applications in healthcare diagnosis and POC analysis of diseases can be detected with such biosensor materials [22]. Carbon allotrope-based nanomaterials consisting of fullerenes, [23,24,25,26,27] nanotubes (CNT) [28,29,30,31,32,33], films of graphene and its derivatives [34,35,36,37], quantum dots [38,39,40,41], and nanodiamonds [42,43,44,45,46,47] play a substantial role in recent advancements in the biosensor domain. In addition to greater sensitivity and novel mechanisms, such sensors offer a higher spatial resolution in case of localised detection along with real-time and label-free non-destructive sensing.

The use of carbon-based nanomaterials for the detection of various biological analytes has been rising over the past decade. In spite of their exceptional material properties, carbon allotrope-based nanomaterials are marred by a deficiency of surface heterogenic reactivity, which is essential for the surface immobilisation of clinically relevant biomarkers. To enhance the biomolecule attachment to the functionalised surface as well as the subsequent protein–protein recognition, precise engineering of nanomaterial interface is required. To this end, a majority of these nanomaterials need to undergo covalent or non-covalent modifications [30,48,49]. A wide array of biomolecules, such as aptamers, enzymes, peptide nucleic acid (PNA), deoxyribonucleic acid (DNA), viruses, antigens, antibodies, ribonucleic acid (RNA), and microRNA can be immobilised on these materials via functionalisation. In all such cases, carbon allotrope-based nanomaterials act as transducers by offering suitable interfaces for the translation of biorecognition inputs to highly sensitive and quantifiable outputs [48]. Although almost all crystalline and amorphous allotropes of carbon have been exploited for healthcare biosensing, this review primarily focuses on sensors incorporating fullerenes, nanotubes, graphene films, quantum dots, and nanodiamonds. 

### 2.1. Fullerene Nanomaterials

**Figure 4:**
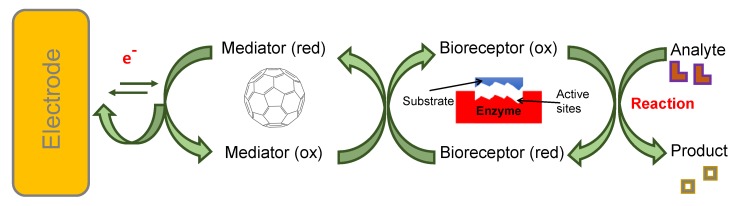
Fullerene as a mediator at the interface of the electrode and the recognition site.

C_60_, the smallest and most common fullerene, is made up of five and six membered sp^2^ hybridised carbon rings forming a truncated icosahedron. In addition to good electrical conductivity and high specific surface area, fullerene shows an excellent biocompatibility, inert behaviour, and structural stability, good affinity towards various organic molecules. It is easy to use and is free from metallic impurities [24,50,51,52]. Since it avoids π bonds in pentagonal rings, leading to inadequate electron delocalisation, C_60_ is not “superaromatic”. Therefore, it possesses superior electron acceptance capacity, enabling it to react with amines and to be decorated with various nanoparticles [25]. Moreover, due to its inner redox activity [53], fullerene can act as an electrophile as well as a nucleophile, allowing its use as a mediator [54] in electrochemical biosensing devices, as illustrated in Figure 4.

Due to poor solubility in aqueous medium, C_60_ tends to aggregate, thus inhibiting its redox activity. This makes the electrochemical behaviour irreversible and unstable in water. This issue can be tackled with covalent functionalisation of fullerene with hydroxyl, carboxyl, or amine groups [25,54]. We can thus obtain fullerenes with tailored properties, such as hydroxyl fullerene, which is water soluble and protects the biological activity of proteins by forming noncovalent complexes [55].

**Figure 5:**
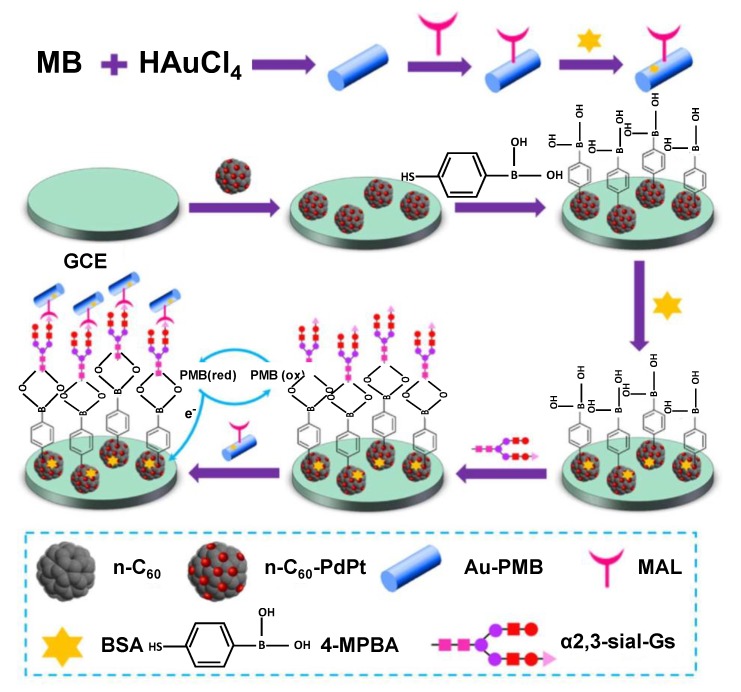
Development of a fullerene–palladium–platinum alloy-based biosensor for the detection of α2,3-sialylated glycans [56].

The properties of C_60_ can also be manipulated by forming nanocomposites with metallic nanoparticles to enhance its loading capacity as well as the electrical conductivity. For example, Yuan and co-workers fabricated a state-of-the-art sandwich-type biosensor for the detection of α2,3-sialylated glycans (α2,3-sial-Gs), a biomarker for early stage cancer diagnosis, using a composite of C_60_ with metallic nanoparticles [56]. The surface of the glassy carbon electrode (GCE) was modified with bimetallic palladium–platinum alloy nanocrystals coupled with amino-functionalised fullerene (n-C_60_-PdPt). 4-mercaptophenylboronic acid (4-MPBA) was immobilised on the n-C_60_-PdPt, as the mercapto functional group can strongly adsorb on PdPt alloy. The coordination of the boron group of the 4-MPBA with the amide group of *N*-acetylneuraminic acid (Neu5Ac) in the structure of α2,3-sial-Gs is responsible for recognition. The redox species consist of Au-poly(methylene blue) (Au-PMB) with covalent immobilisation of maackia amurensis lectin (MAL). A schematic representation of the experimental protocol is illustrated in Figure 5. The current response of the sensor was obtained with the help of differential pulse voltammetry (DPV). The sensor harnessed the excellent electron transfer capacity afforded by fullerene and its large surface area for the in-situ reduction of PdPt alloy nanocrystals. The sensor was effective over a broad concentration range of 10 fg mL^−1^−100 ng mL^−1^, with a very low detection limit (LOD) of 3 fg mL^−1^ [56].

Another approach involved a molecularly imprinted polymer (MIP, synthetic antibodies)-based sensor using functionalised C_60_ as a monomer [57]. Vinyllic-C_60_-monoadduct was allowed to self-assemble in a sodium dodecylsulfate (SDS) micellar system to obtain neoteric water-compatible C_60_-monoadduct in the presence of chlorambucil template (anti-cancer drug). The polymerisation of the fullerene monoadducts was performed with ammonium persulfate as an initiator and *N*,*N*-methylenebisacrylamide as a crosslinker. Following template removal by dynamic incubation in acetonitrile, the immobilisation of imprinted micellar nanoparticles on the surface of ionic liquid decorated ceramic carbon electrode (IL-CCE) was performed. This was possible due to the π–π interactions between the sp^2^ π electron cloud of fullerene and IL-CCE. A perfectly linear current-concentration profile was obtained in the range of 1.47–247.20 ng mL^−1^ and the limit of detection was found to be 0.36 ng mL^−1^ in real and aqueous samples. This study utilised fullerene as a nanomediator and a four-fold transduction was realised in comparison to an electrode with no C_60_-monoadduct immobilisation when evaluated by differential pulse anodic stripping voltammetry (DPASV) and cyclic voltammetry (CV) [57]. Mazloum-Ardakani and co-workers used a similar ionic liquid by replacing MIPs with antibodies to generate a label-free electrochemical immunosensor to detect tumour necrosis factor α (TNF-α) [50]. They modified graphite screen printed electrodes (GSPE) with a nanocomposite of fullerene and multiwalled carbon nanotubes (MWCNT) to entrap anti-TNF-α. The GSPE-C_60_-MWCNT-IL-anti-TNF-α immunosensor demonstrated a detection range of 5.0–75 pg mL^−1^ with a low detection limit of 2.0 pg mL^−1^.

Rather et al. exploited the electron affinity of C_60_ nanorods (C_60_NRs) to covalently add N–H from nitrophenylene modified GCE (GCE–Ph–NH_2_) across its π bond to obtain an electrochemical sensor for the detection of paraben (endocrine disruptor) [26]. Following electrochemical reduction, a highly conductive sensor (ERC_60_NRs–NH–Ph–GCE) was obtained. This sensor was capable of detecting ethylparaben (EP) from 0.01 to 0.52 μM with an LOD of 3.8 nM. Electrochemical impedance spectroscopy (EIS) and CV were used to study the electrocatalytic activity of the sensor [26]. Demirbakan and Sezgintürk used C_60_ as an immobilisation surface on GCE to link heat shock protein 70 antibody (anti-HSP70) with the help of an 1-Ethyl-3-(3-dimethylaminopropyl)-carbodiimide/N-hidroxysuccinimide (EDC-NHS) mixture in order to fabricate a novel immunosensor for sensitive HSP70 detection [51]. This GCE-C_60_-anti-HSP70 sensor, using CV and EIS as measurement methods, could successfully detect HSP70 concentrations between 0.8 and 12.8 pg mL^−1^. Barberis et al. employed a combination of fullerene and graphite to generate sensor–biosensor systems (SBs) for ascorbic acid (AA) recognition [52]. They coupled ascorbate oxidase (AOx) with the biosensor and made a comparison between C_60_-modified and C_70_-modified graphite. The investigation range evaluated using amperometric detections for the study was 0–20 μM. The detection limit was found to be 0.10 μM and 0.13 μM for SBs with C_60_ and C_70_ modifications, respectively. The C_60_ modification enhanced the sensitivity of graphite electrode 1.2 times, whereas the C_70_ increased it by 1.5 in comparison to unmodified graphite. The selectivity of fullerene-modified SBs was superior to that of nanotube-modified SBs, since fullerenes are capable of holding more AOx. Another study, coupling an enzyme with a biosensor system, involved the fabrication of an electrochemical glucose biosensor, where C_60_ was used as a moderator for the direct electron transfer of glucose oxidase (GOx) on reduced graphene oxide (RGO) [58]. CV and amperometry *i-t* were able to obtain a linear response for glucose detection in a concentration range of 0.1–12.5 mM with an LOD of 35 μM. A similar study for glucose biosensing replaced RGO with GCE and C_60_ with hydroxyfullerene (HF) to promote linking and complex formation with GOx [55]. The GOx–HF nanocomposites were immobilised on GCE and protected with a membrane of chitosan (chit). CV and linear sweep voltammetry (LSV) proved that the GCE–GOx–HF–chit sensor was functional to measure glucose from 50 μM to 1.0 mM with an LOD of 5 ± 1 μM. Shahhoseini et al. recently developed a non-enzymatic glucose biosensor by coating GCE with the Ni (II) one-dimensional coordination polymer of methyl pyridine (pMPy) (Ni(II)-pMPy) and C_60_ [59]. The C_60_ nanocomposite biosensor was able to detect glucose in the concentration range of 0.01–3 mM with a limit of 4.3 μM. Additional examples of fullerene and other carbon allotrope-based electrochemical biosensors are summarized in Table 1.

### 2.2. Carbon Nanotubes

CNTs are regarded as auspicious building blocks of biosensors as a result of their high aspect ratio, large surface area, excellent thermal and chemical stability, superior mechanical strength, and exceptional optical and electronic properties [18]. CNTs offer an edge to biosensors due to their high sensitivity, excellent signal-to-noise ratio, low background, broad absorption spectrum, label-free detection, and real-time monitoring [22]. They serve as scaffolds for biomolecule immobilisation, thereby ameliorating signal transduction and subsequently recognition [74]. The semiconducting nature of carbon nanotubes allows their use as nanoscale field effect transistors (FET) [32]. They can be used to manufacture top-of-the-line nanoscale electrodes due to the superior excellent conductivity along their length. CNTs have exceptional wavelength conversion function, particularly the adjustable near-infrared emission, which represents fluctuations in local dielectric function but is resistant to permanent photobleaching. In addition, they show high luminous intensity and excellent luminous properties, which are ideal for optical biosensing [75,76,77,78,79,80]. CNTs have only a sixth of the density of steel but are 100 times stronger, allowing their use in fabricating piezoresistive sensors [81,82,83,84]. It is also possible to synthesize CNT-based calorimetric sensors which rely on changes in the size of nanotubes caused by thermal variations [18,85].

Numerous CNT-based biosensors for glycaemic biomarkers of diabetes mellitus have been recently reported. Hatada et al. reported a label-free chemiresistor-type FET affinity sensor for haemoglobin A1c (HbA1c) using single-walled carbon nanotubes (SWCNT) as a transducing element and a bacterial periplasmic protein (SocA) as a receptor [32]. HbA1c, on proteolytic hydrolysis, produces fructosyl valine (FV) that could be quantified by the sensor in a concentration range of 1.2–1909 nM. Comba and co-workers developed a durable enzymatic biosensor for glucose using a mucin–CNT (CNT-muc) nanocomposite immobilised on a platinum surface [86]. The large surface area of CNT promoted the immobilisation of GOx enzyme. The Pt–CNT–muc–GOx sensor could sense glucose in a range of 0.002–3.2 mM by employing chronoamperometry and the LOD was found to be 3 μM. Another study eliminated the requirement of the GOx by fabricating MWCNT scaffolds with cobalt functionalised MoS_2_ [87]. This scaffold was suitable for glucose sensing over a linear concentration range of 0.2–16.2 mM with an extremely low detection limit (80 nM). Aryal and Jeong reported a thermally reduced graphene oxide–MWCNT (TRGO-MWCNT) nanocomposite modified with ambient plasma and β-cyclodextrin (βCD) for uric acid (UA) detection [88]. The LOD of this sensor was 0.06 µM and the sensor could provide linear responses from 10 µM to 300 µM. Bollella et al. exploited the exceptional electron exchange properties between MWCNT and poly(methylene blue) (pMB) to develop the first second-generation Au microneedle sensor for continuous lactate detection in dermal interstitial fluid [89]. Lactate detection between 10 and 200 µM was possible following lactate oxidase (LOx) immobilisation on the sensor and a very low detection limit (2.4 µM) was obtained. Shen et al. reported a chemiresistive paper-based label-free immunosensor for cost-effective POC detection using SWCNT [33]. They harnessed the non-covalent π–π stacking interactions between SWCNT and pyrene carboxylic acid (PCA) to synthesise a water-based ink prior to human serum albumin (HSA) antibody immobilisation. The SWCNT–PCA–antiHSA ink was able to sense 0.015–9.43 nM HSA and demonstrated an LOD of 1 pM.

Huang and co-workers developed an immunochromatographic assay which enabled visual evaluation of rabbit immunoglobulin G (IgG) using goat anti-rabbit IgG antibodies (Ab_1_) immobilised on MWCNT magnetised with Fe_3_O_4_ (MMWCNT) [80]. Visual detection in blood revealed a detection limit of 10 ng mL^−1^ and a linear dynamic range between 10 and 200 ng mL^−1^. Another visual immunochromatographic biosensor, capable of detecting carcinoembryogenic antigen (CEA), a lung cancer biomarker, was manufactured using a CNT-gold nanoparticle (AuNP) nanocomposite with detection antibody (dAb) coating as a reporter probe [75]. The cotton thread-based device provided a direct readout by the naked eye with a linear response in the range of 10–500 ng mL^−1^ and LOD of 2.36 ng mL^−1^. Meng et al. reported a similar optical biosensor for another lung cancer biomarker, human ferritin antigen (HFA), using MWCNT to obtain a linear concentration range from 100 to 5000 ng mL^−1^ with LOD of 50 ng mL^−1^ [76]. Optical sensors based on surface plasmon resonance (SPR) using CNT have also been reported. Pathak and Gupta developed a polypyrrole (PPy) MIP on carboxylated multi-walled carbon nanotubes (CMWCNT) with a permselective nafion membrane for the SPR detection of dopamine (DA) [77]. This sensor demonstrated a wide dynamic range of 10^−9^–10^−5^ M with an LOD of 18.9 pM in synthetic cerebrospinal fluid. Another SPR sensor employed a radically different strategy to detect human cardiac troponin I (cTnI) using Fe_3_O_4_@PDA-dAb as immune probes on a capture antibody (cAb) immobilised Au platform, which was coated with polydopamine (PDA) and modified with AuNPs [78]. In this study, MWCNTs were used in conjunction with silver nanoparticles (AgNPs) and PDA with secondary antibody decoration (Ab_2_) to enhance the SPR response. Lee et al. developed a novel plasmon-assisted fluoro-immunoassay (PAFI) for quantification of influenza virus H3N2, relying on AuNP decorated MWCNT nanohybrids with immobilised virus antibodies (Abs) [79]. A low detection limit of 50 plaque forming units per mL (pfu mL^−1^) was obtained and the sensor provided a linear response between 50 and 10,000 pfu mL^−1^.

**Figure 6:**
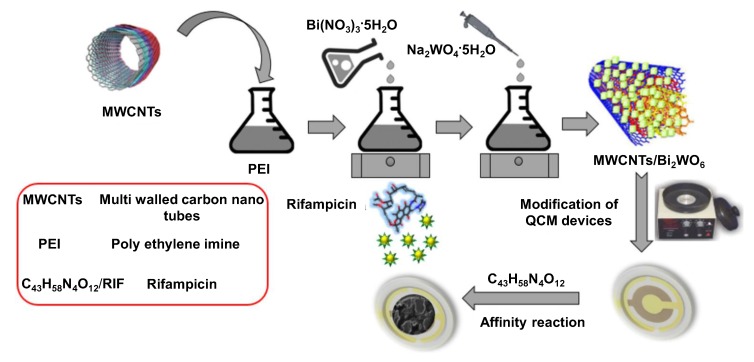
Scheme for fabricating a quartz crystal microbalance (QCM)-based rifampicin sensor using multiwalled carbon nanotubes (MWCNT) and Bi_2_WO_6_ [90].

Interdigital electrodes (IDE) of series piezoelectric quartz crystal (SPQC) have been frequently coupled with CNTs to develop piezoelectric sensors for clinical diagnosis [81,82,83,84]. Zhang et al. developed an aptasensor in this way for the detection of *Mycobacterium tuberculosis* (H37Rv) with the help of a single-strand deoxyribonucleic acid (ssDNA) aptamer [81]. The sensor provided a linear signal in the concentration range of 1 × 10^3^–1 × 10^7^ cfu mL^−1^ with an LOD of 100 cfu mL^−1^. In another study, a lysozyme (LZM) aptasensor exploiting the electron transfer between SWCNT and an IDE of series piezoelectric quartz crystal (SPQC) was reported to generate linear frequency shifts from 1 to 80 nM of LZM with a detection limit of 0.5 nM [83]. A proof-of-concept mass sensitive detection of rifampicin, a tuberculosis drug, using a nanocomposite of MWCNT and Bi_2_WO_6_ as an affinity material for quartz crystal microbalance (QCM) is illustrated in Figure 6. The sensor demonstrated a linear response in the range of 1–700 μM and achieved an LOD of 0.16 μM [90].

CNT biosensors are commonly used for the detection of various cancer types and neurological disorders. For an instance, a chemiresistive paper-based CMWCNT biosensor was constructed for prostate specific antigen (PSA), a common prostate cancer biomarker. This antibody sensor allowed the quantification of the biomarker down to 1.18 ng mL^−1^ [91]. Another antibody-based CNT sensor for the recognition of P-glycoprotein (P-gp), a leukemia biomarker, employed anti-P-gp-SWCNT film developed on a SiO_2_–Si substrate. The sensor could assay 1.5 × 10^3^–1.5 × 10^7^ cells mL^−1^ with an LOD of 19 cells mL^−1^ [92]. Keihan et al. proposed a bamboo like MWCNT (BCNT) nanocomposite with ionic liquid (IL) and Prussian Blue (PB) for the enzymatic determination of choline. With this sensor, a linear calibration was possible in between 4.5 × 10^−7^ and 1.0 × 10^−4^ M [93]. In a similar study for the enzyme-based quantification of serotonin, a monoamine neurotransmitter associated with memory and happiness, a GCE was functionalised with MWCNT with monoamine oxidase A (MAO-A) immobilisation. The potentiometric detection of serotonin in a concentration range of 5.67 × 10^−7^–2.26 × 10^−6^ M was achieved in simulated body fluid with a high sensitivity (LOD: 2 × 10^−7^ M) [94].

### 2.3. Graphene and Graphene Derivatives

Graphene is a relatively recent type of carbon allotrope made up of sp^2^ hybridised carbon atoms assembled in a hexagonal configuration. The electrons in graphene impart unusual properties, such as ambipolar electric field effects, excellent thermal conductivity, and quantum hall effects at room temperature. It has a 2D structure, giving rise to extremely high surface area and high porosity. This makes graphene suitable for the adsorption of various gases, such as methane, hydrogen, and carbon dioxide [2]. Properties of graphene can be tuned by manipulating the number of layers and the stacking order. It is highly transparent with a strong resistance to fracture and a high modulus of elasticity. In addition, graphene is capable of interacting with various biomolecules via physisorption, making it an ideal candidate for biosensors [18]. Graphene derivatives can display interesting properties, including graphene oxide (GO), which exhibits fluorescence. GO, RGO, and graphene quantum dots (GQDs) are the most essential graphene derivatives employed in the biosensing field [95,96]. Graphene-based biosensors are highly versatile and can be used for the detection of cysteine [70,97,98,99,100], glycaemic biomarkers [58], cholesterol [2], neurotransmitters [60,64], H_2_O_2_ [101], cancer cells [102], nucleic acids [2], pharmaceutical drugs [39], and infectious bacteria [96].

Graphene has been used in the fabrication of many optical and electrochemical biosensors for the detection of various amino acids. Kumar et al. exploited the large surface area of electrochemically-reduced graphene oxide (ERGO) by chelating it with a complex of 1,10-phenanthroline-5,6-dione(phen-dione) and Cu^+2^ to obtain a composite GCE for cysteine (Cys) detection [97]. This sensor presented a linear response between 10.0 and 32,344.0 μM with a detection limit of 2.0 μM. In another study, a bifunctional optical sensor based on the photophysical properties of AuNP decorated GQD was used for the determination of lysine and Cys [98]. The sensor could determine Cys and Lys in the concentration ranges of 0.05–0.5 mM and 0.047–0.8 mM, respectively. The corresponding LODs were found to be 16.14 μM and 5.88 μM. 

Graphene-based biosensors play a pivotal role in the sensing of incurable and fatal diseases, such as diabetes and various kinds of cancer. Jaberi et al. developed an RGO–Au nanostructure paper-based electrochemical nano-genosensor for HbA1c determination on a flexible and cost-effective graphite sheet (GS) electrode [103]. A wide concentration of 1–13.83 μM could be successfully determined by the thiolated aptamer-based genosensor. The amount of HbA1c can be affected by various diseases, such as sickle cell anaemia, haemolytic anaemia, and haemoglobinopathy, making it an unreliable biomarker for conclusive diagnosis of diabetes mellitus. Apiwat and co-workers overcame this issue by substituting HbA1c with glycated HAS (GHSA) as a biomarker [104]. They immobilised biotinylated aptamer on fluorescent quenching GO and obtained a linear response between 0.05 and 0.3 mg mL^−1^ and a detection limit of 50 μg mL^−1^. A study on LZM detection reported a state-of-the-art ultrasensitive aptamer-based chemiluminescent sandwich biosensor using a GQD–GO–carbon fibre (CF) nanocomposite with an LOD of 12.5 pg L^−1^ and a wide linear range of 2.64 × 10^−10^–6.6 × 10^−8^ g L^−1^ [105]. Shahrokhian and Salimian developed a highly sensitive genosensor for detecting BRCA1, a genetic biomarker of breast cancer, using a GCE–ERGO electrode with poly (pyrrole-3-carboxylic acid) (PP3A) and E-DNA functionalisation [102]. The GCE–ERGO–PP3A–E–DNA electrochemical sensor could successfully detect the biomarker in the concentration range of 10 fM–0.1 µM BRCA1 with a detection limit of 3 fM. Dong et al. succeeded in detecting H_2_O_2_ from living breast cancer cells using physisorption of Au–Pd bimetallic nanocubes (AuPdNCs) and RGO on GCE [101]. Due to the high electroactive surface area and exceptional electrical conductivity, the sensor displayed a low detection limit of 4 nM and a broad linear response in the range of 0.005 μM–3.5 mM. Diao and co-workers monitored propofol, an intravenous anaesthetic agent, in the presence of horseradish peroxidase (HRP) and H_2_O_2_ using fluorescent GQDs derived from the carbonisation of citric acid [39]. The optical sensor determined propofol in a range of 5.34–89.07 mg L^−1^ with an LOD of 0.5 mg L^−1^.

### 2.4. Carbon Quantum Dots

Quantum dots are zero-dimensional semiconducting particles with a size of a few nanometres [96]. They have exceptional optical and fluorescent properties. Carbon-based quantum dots are classified into crystalline GQDs and amorphous carbon dots (CD). As GQDs were discussed along with other graphene biosensors in Section 2.3, this section focuses on CDs. In comparison to GQD, CDs are more water soluble and show a superior biocompatibility due to a greater number of carboxylic moieties on their surface [106]. Easy functionalisation with polymers, biomolecules, and various other organic and inorganic molecules as well as surface passivation can enhance their fluorescence and physical properties, making them ideal candidates for medical diagnostics and bioimaging [107]. CDs have also been used in association with other materials to fabricate biosensors with enhanced sensitivity, reliability, and rapidity [2].

CDs have gained extensive scientific interest as the material of choice for the detection of a variety of cancer biomarkers for early stage diagnosis, as well as monitoring tumour proliferation. Abdelhamid and co-workers developed a gold doped CD–cytosensor for the recognition of metallic ions, such as Fe^3+^, in cancer cells [108]. The Au–CD nanocomposite displayed a maximum absorbance of 337 nm, making it an ideal candidate for surface enhance laser desorption/ionisation mass spectrometry (SELDI-MS), since the wavelength of an N_2_ laser is 337 nm. The sensor was capable of detecting Fe^3+^ when chelated with mefenamic acid (MFA) in cancer cells within the range of 1.0–300.0 nM, following cell separation [108]. Another sensor for Fe^3+^ detection employed metal-free nitrogen doped CDs [40]. Intrinsic properties of CDs can be modulated by such doping methods. The fluorescent sensor was active for Fe^3+^ in a concentration range of 5–20 μM with an LOD of 3.7 μM. 

CEA detection by optical aptasensors using nitrogen and sulphur co-doped CDs (NS-CD) as well as undoped CDs has been reported. The former study reported a lower LOD of only 94 pg mL^−1^ with a broad linear response in 0.3–80 ng mL^−1^, likely due to the HRP-AgAu-aptamer label [109]. In the case of the label-free undoped CD aptasensor, a narrower detection range between 0.5–1 ng mL^−1^ with a lower sensitivity (LOD: 0.3 ng mL^−1^) was obtained [110]. Mohammadi et al. developed a fluorescence resonance electron transfer (FRET)-based immunoassay for the cancer antigen 15-3 (CA 15-3), a breast cancer biomarker, using a sandwich assay of CD-anti CA15-3 with polyamidoamine (PAMAM) dendrimer and CA 15-3 aptamer co-immobilised AuNPs as the redox probe [111]. The detection range of this sensing assembly was 1.1 μU mL^−1^–5.0 mU mL^−1^, with an LOD of 0.9 μU mL^−1^.

In addition to cancer, CDs have been used for the diagnosis of other fatal and incurable diseases. Liang and co-workers developed a ratiometric fluorescence (RF) assay using CDs and cadmium telluride (CdTE) quantum dots (QD) for the detection of HIV DNA [112]. Mitoxantrone (MTX) was used as a fluorescence quencher for CdTeQDs. A linear detection was achieved from 1.0 to 50.0 nM of DNA concentration. Cho and Park reported an RF sensor using CDs and rhodamine 6G (Rh6G) with immobilised GOx and HRP for glucose detection [113]. In aqueous solution, the sensor provided a linear photoluminescent response between 0.1 and 500 µM with an LOD of 0.04 µM and a good selectivity in the co-existence of many non-specific molecules in blood. The detection of volatile organic biomarkers (VOBs) is a reliable, rapid, inexpensive, and portable method for medical diagnosis. Bhattacharya et al. reported a fluorescent CD biosensor for tuberculosis (TB)–VOBs using breath analysis techniques [114]. They mixed the CDs with methyl nicotinate dissolved in ethyl alcohol and then directed TB-VOBs bubbles through a methyl nicotinate solution with a nitrogen flow onto the aforementioned sensor. The sensor succeeded in detecting up to 10 mM of TB-VOBs. The lowest concentration investigated in this study was 2 mM.

### 2.5. Nanodiamonds

In comparison to the carbon allotrope-based nanomaterials previously discussed, nanodiamonds (NDs) are the only ones comprised of sp^3^ hybridised carbon centres. NDs represent outstanding properties of bulk diamond, such as wide band gap electronic behaviour, chemical inertness, thermal conductivity, and exceptional mechanical properties to those derived by their high specific area, which can reach 400 m^2^ g^−1^ [115]. They can be synthesised by grinding microdiamonds under high pressure high temperature (HPHT) conditions or by the detonation of carbonaceous explosives (DND). They can link covalently or non-covalently with biomolecules after simple functionalisation with amines, thiol group halides, or hydroxyl groups. HPHT diamonds are rich in nitrogen impurities, which are amenable to transformation into vacancy-related colour centres, thus forming fluorescent nanodiamonds (FND). The photophysical characteristics of the vacancy centres allow FNDs to act as efficient biosensing, as well as bioimaging probes and contrast agents [116,117,118].

NDs have been extensively used in biosensing due to their fluorescent nature and their ability to detect a variety of metal ions. Shellaiah and co-workers developed photoluminescent cysteamine (CYA)-modified nanodiamonds for the detection of Hg^2+^ ions [119]. CYA forms amide linkages with NDs and possesses free thiol groups capable of trapping mercury ions. The sensor provided a linear response to Hg^2+^ samples from 100 nM to 100 µM and revealed an LOD of 153 nM. Instead of functionalisation, NDs can also be doped with nitrogen for the quantification of heavy metal ions. Monodimensional nitrogen doped nanodiamond nanorods (*N*-DNR) were used as an electrochemical sensor for detecting Pb^2+^ and Cd^2+^ simultaneously. CV and square wave anodic stripping voltammetry (SWASV) measurements resulted in a detection range of 0.05–1 µM and 0.01–1.1 µM for Pb^2+^ and Cd^2+^, respectively. The LODs were found to be 0.05 µM and 0.01 for the corresponding heavy metal ions [120].

**Figure 7:**
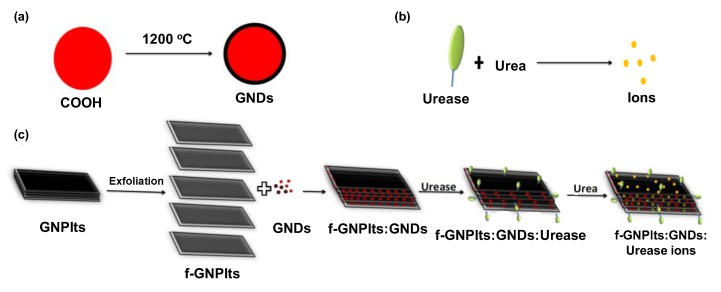
Scheme depicting sensing mechanism using f-GNPtlts-GNDs: (**a**) graphitisation of nanodiamonds, (**b**) urea hydrolysis in presence of urease, (**c**) sensor fabrication and urea detection [121].

Various clinically relevant compounds, including neurotransmitters, pharmaceuticals, and toxins, as well as biomarkers for chronic illnesses such as diabetes, have been detected using NDs [121]. Dai and co-workers electrophoretically deposited NDs on a boron doped diamond (BDD) electrode and subsequently modified it with Ni nanosheets for enzyme free detection of glucose [122]. The electrochemical sensor was functional for measuring glucose in a concentration range of 0.2–1055.4 μM, which provided an LOD of 50 nM. Briones et al. developed a GOx-based glucose sensor prototype to fabricate the first electrochemical nanodiamond lactate biosensor on a gold electrode platform [123]. They substituted GOx with LOx. The neoteric sensor provided a detection range of 0.05 to 0.7 mM and a detection limit of 15 µM. An enzymatic CNT screen printed electrode (CNT-SPE) with a nanocomposite of carboxyl functionalised graphene platelets and graphitised nanodiamonds (f-GNPtlts-GNDs) was reported to successfully detect urea down to 5 μg mL^−1^ (Figure 7) [121]. Peltola et al. enhanced dopamine detection using hydroxyl functionalised nanodiamonds (ND_H_) integrated with tetrahedral amorphous carbon (ta-C) thin films [47]. They made a comparison between variously functionalised NDs and showed that ND_H_ addition to the thin film electrodes lowered the detection limit of the sensor by two orders of magnitude, from 10 µM to 50 nM. The potentiometric method was capable of quantifying dopamine from 50 nM to 1 mM using ND_H_ integrated ta-C films. Simioni et al. constructed an ND-GCE sensor for the detection of pyrazineamide (PZA), an antibiotic for tuberculosis treatment that has serious side effects in cases of overdoses [124]. The electrochemical sensor was functional up to 4.9 × 10^−5^ M of PZA. In addition to electrochemical biosensors, carbon allotrope-based nanomaterials find abundant applications in optical, piezoelectric, and other types of biosensors, as shown in Table 2. Excluding the aforementioned carbonaceous nanomaterials, nanoonions [125,126], nanohorns [127,128], and many other carbon allotropes have started to emerge as interesting materials for biosensing applications [2,18,22].

## 3. Applications of Inorganic Nanomaterials in Healthcare Biosensing

Transitional metals and noble metals display extraordinary properties at the nanoscale. The excess surface atoms coupled with incompletely filled penultimate or pre-penultimate orbitals give rise to unique quantum effects and optical properties. They can not only form good alloys but can also be used in combination with various organic and carbon-based materials to form nanocomposites, exhibiting a combination of different characteristics or entirely new qualities [2]. Inorganic nanomaterials may possess different anisotropies, such as triangular, spherical, and nanohole [133]. They appear in different forms, such as bimetallic alloys, core-shell structures, metal organic framework (MOFs), nanotube, and nanowire arrays [56,67,71]. Each of these nanomaterials is capable of enhancing the biocompatibility and transduction characteristics of biosensors with the help of appealing interface and surface features. They may act as immobilisation platforms, enhance refractive index changes, catalyse reactions between substrates and chemiluminescents, magnify mass changes, and accelerate electron transfer [134,135,136,137,138,139,140,141,142]. In electrochemical sensors, such nanoparticle platforms may also act as electron wires apart from immobilisation, and thus transduce the biomolecular physicochemical changes to quantifiable signals.

Some inorganic nanomaterials, such as Fe_3_O_4_, demonstrate a magnetic nature and can be easily manipulated by an external magnetic field, allowing simple extraction and buffer replacement, and also provide a high signal-to-noise ratio in biological samples, in addition to their large surface area [143,144,145,146]. They can be used for homogenising, trapping, enriching, transporting, and labelling of analytes, especially in POC testing. They can be applied for microfluidic mixing, which is crucial to lab-on-chip biosensing. Most magnetic nanoparticles (MNPs) consist of a magnetic core of pure elements (like Co and Fe), alloys (like FePt), or iron oxides (such as maghemite γ-Fe_2_O_3_ or Fe_3_O_4_). This core is generally coated with inorganic [147] or polymeric [148] molecules, which act as sites for biofunctionalisation. Embedding multiple MNPs in a non-magnetic matrix may result in superparamagnetic behaviour [2,143].

Novel inorganic architectures such as nanoshells, nanocages, and nanowires have recently gained much attention for biosensor development. Nanoshells, usually comprising a dielectric silica core enveloped in a highly conducting, ultrathin layer of silver or gold, constitute a new class of nanomaterials with tunable plasmon resonance, permitting materials to be particularly engineered to match the wavelength for specific applications, such as near infrared (NIR) areas where optimal light penetration through tissue is required [2,149]. Nanoshell substrates with surface enhanced Raman spectroscopy (SERS)-based sensors are promising platforms for in vivo detection [150,151,152]. Nanocages are nanostructures with hollow interiors and porous walls, usually made of noble metals [69,153]. Due to their high surface area, they show great potential for bio-functionalisation and biomolecule immobilisation. One-dimensional (1D) nanowires typically possess a length:diameter ratio above 1000 and may be semiconducting or dielectric in nature. They exhibit unique electrical and thermal properties. Noble metal nanowires demonstrate localised SPR (LSPR)-like properties that can be tuned according to their thickness [154,155,156]. Nanowire arrays can penetrate cellular lipid bilayers like nanoneedles, enabling cytosensing and similar medical diagnostic uses. Silicon oxide NW can act as a substrate for receptor immobilisation in FET biosensors [2,155].

A wide variety of inorganic nanomaterials have been used in healthcare diagnostics. In this review we mainly focus on the commonly utilized nanomaterials, including quantum dots, magnetic nanoparticles, noble metal nanoparticles, and nanostructures such as nanoshells, nanowires, and nanocages.

### 3.1. Quantum Dots

Inorganic QDs generally consist of a bimetallic alloy core with a shell layer, such as metal chalcogenide. The dominance of quantum confinement effects takes place when the diameter of QDs is less than the electron-hole Bohr radius, giving rise to unique optical properties. Stokes shifts, arising from the NIR or UV electromagnetic radiation during excited electron relaxation to holes, cause superior photoluminescence in comparison to organic dyes. QDs have been used for the detection of a variety of molecules, such as proteins [157,158], pathogens [159], lung cancer biomarkers [160], and nucleic acids [161,162]. Nevertheless, widespread in vivo use of quantum dots is still inhibited by the toxicity of cadmium, a common constituent in QDs, as well as by the tendency of protective coatings to undergo in vivo degradation [163].

**Figure 8:**
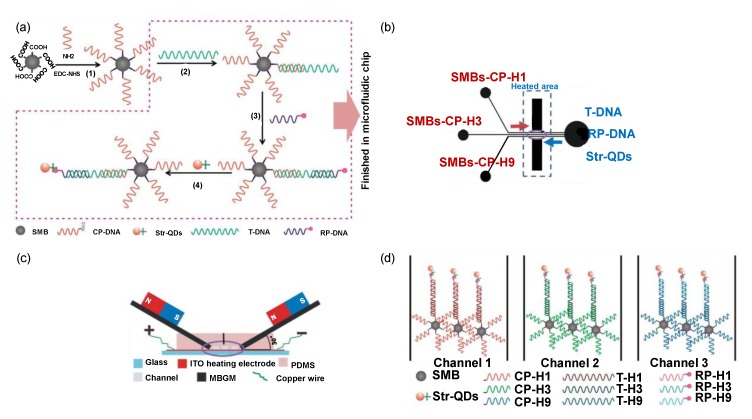
(**a**) Experimental steps for developing quantum dot-based microfluidic chips for virus detection. (**b**) Schematic representation of the biosensor and sample loading process. (**c**) Cross-section of the microfluidic chip. (**d**) Principle of multiple viruses recognition and simultaneous subtyping on the microfluidic device [164].

A wide array of target molecules can be effectively detected by implementing QDs in a micro-fluidic platform. Recently, such a sensor was used for the determination and subtyping of three influenza viruses (H1N1, H3N2, and H9N2) [164]. Streptavidin-coated quantum dots (Str-QDs) with immobilised biotinylated DNA were used as labels for fluorescent imaging and DNA immobilised superparamagnetic beads (SMB) acted as capture probes (Figure 8a). The sensor operation was based on the hybridisation of nucleic acid on the microfluidic chip in the presence of a regulated micro-magnetic field (Figure 8c,d). The simultaneous detection of H1N1, H3N2, and H9N2 was performed in 80 min in the ranges of 1–150 nM, 5–150 nM, and 1–150 nM with LODs of 0.21 nM, 0.16 nM, and 0.12 nM, respectively [164]. A similar study for the detection of common peanut allergen (Ara h1) was reported using QD–aptamer–GO hybrids [165]. This system acted as a probe that underwent conformational changes due to the adsorption and desorption of GO on biotinylated aptamer functionalised Str-QDs. Within 10 min, the microfluidic sensor provided a single step homogeneous assay. The miniaturised optical sensing system was functional in a concentration range of 200–2000 ng mL^−1^ with an LOD of 56 ng mL^−1^ [165]. The sensing method thus exhibited a potential for on-site allergen detection.

Another study on protein recognition reported the fluorescence quenching of CdSe quantum dots using metal oxide nanoparticles of Eu_2_O_3_ and CuO, as well as noble metal nanoparticles of silver and gold [157]. Following the addition of analyte, the fluorescence activity of QDs was restored, as the analyte–QD interactions liberated QDs from the nanoparticle–QD conjugate. The interactions between the proteins and the QDs also improved the fluorescent intensity. The LODs in all cases were below 2 μM. CuO–CdSeQDs and Eu_2_O_3_–CdSeQDs recognised casein in the ranges of 0.5–5 μM and 2.0–50 μM, respectively [157]. AgNP–CdSeQDs were successful in papain detection from 3.0 to 20 μM and the AuNP–CdSeQDs provided a linear response between 2.0 and 50 μM. DA functionalised CdSe–ZnS QDs have been investigated for the detection of α-fetoprotein (AFP), an important biomarker for various kinds of tumour and prenatal aneuploidy, with an immunoassay based on redox-mediated indirect fluorescence [158]. Tyrosinase(TSA)-detection antibody conjugate acted as bridges between biomarker concentrations and QD signals by catalysing the DA oxidation on the QD surface. AFP detection was reported between 10 pM and 100 nM and the detection limit was 10 pM. Specificity of 100% and 97.5% sensitivity, with a clear distinction between positive and negative samples, was observed when the sensor system was employed to validate AFP detection in 10 different AFP-negative control samples and 40 AFP-positive samples obtained from hepatocellular carcinoma patients. A combination of QD fluorescence and immunomagnetic separation (IMS) has been reported to hold promising results in pathogen detection [159]. Magnetic nanoparticle cores with gold shells, functionalised with biotinylated *E. coli* antibodies, were employed as capture probes for *E. coli* and chit-coated CdTeQds acted as reporter probes in a sandwich immunoassay. The bacteria were extracted from the sensing solution using IMS prior to fluorescence analysis. The sensor showed a low cross-reactivity against non-specific bacteria and achieved a detection limit of 30 cfu mL^−1^ with a wide linear concentration range of 10^2^–10^8^ cfu mL^−1^. Wu et al. employed this strategy by using multicoloured QDs for the detection of the lung cancer biomarkers with carboxyl functionalised micro-magnetic beads (CMMB) acting as immune carriers [160]. Fluorescence measurements revealed a successful detection of CEA, cytokeratin-19 fragments (CYRFA-21), and neuron-specific enolase (NSE), with LODs of 38 pg mL^−1^, 364 pg mL^−1^ and 370 pg mL^−1^, respectively.

MicroRNA or miRNA is a vital biomarker for early stage diagnosis of cancer. Therefore, sensitive and rapid determination of miRNA is important for POC testing. Deng and co-workers fabricated a strip biosensor labelled with QDs that quantified miRNA-21 [161]. The photostable QDs improved the detection efficacy of the biosensor. The sensitivity of the system was further enhanced using a target-recycled amplification strategy based on sequence-specific and enzyme-free hairpin strand displacement mechanism. The sensing platform was functional in a range of 2–200 fmol, with a limit of 200 amol. The analysis was comparable with results obtained from real-time polymerase chain reaction (PCR). In addition, 16 out of 20 clinical tumour samples provided positive signals. Another similar study enhanced the resonance light-scattering (RLS) intensity of cadmium tellurium quantum dots (CdTeQDs) with the help of a hybrid mixture of cDNA probes (CDTEQD-P) [162]. The CdTeQD-Ps exhibited low intensity in the absence of miRNA-122 and coexisted stably in the solution. However, they formed proportionate aggregates by complexation with miRNA-122 with increased intensity. The enhancement in the RLS intensity could be observed for concentrations between 0.16 and 4.80 nM, with a low limit of 9.4 pM. Such fluorescence properties of metallic nanoparticles have been extensively harnessed for the construction of optical biosensors (Table 3).

### 3.2. Magnetic Nanoparticles

Fe_2_O_3_, Fe_3_O_4_, FePt, and many other similar nanoparticles are superparamagnetic in nature. Depending on their synthesis mechanism, these particles may have varying size distributions and their sizes range from 10 nm to 1000 nm. MNPs are generally employed as either transducers, which may be electrochemical, piezoelectric, optical, or colorimetric in nature, or as labels in conjugation with biomolecules [146]. MNP biosensors are useful in various disciplines, including the food industry, medical diagnosis, and environmental investigations [177]. There are three prerequisites that need to be considered for utilising MNPs in healthcare biosensing: (i) MNPs must retain a high saturation magnetisation to enable the manipulation of their movement in blood without the requirement of very strong magnetic fields. allowing the movement of MNPs in close proximity to the targeted tissue; (ii) MNPs must be biocompatible and non-toxic; (iii) the size of MNPs should range between 10 and 50 nm to avoid aggregation or precipitation owing to gravitational forces and to ensure the colloidal stability, especially in water at pH 7.0, thereby yielding a large surface area for a specific volume of the material [146,178,179].

The application of MNPs in early stage cancer sensing has exhibited tremendous potential. For example, Pal et al. multiplexed MNPs with monoclonal antibodies (mAbs) for sensing various ovarian cancer biomarkers (cancer antigen 125 (CA-125), Apo-lipoprotein A1 (ApoA1), and β2-microglobulin (β2-M)) [180]. A sandwich assay was developed with the help of polyclonal antibodies (pAbs). The sandwiched particles were subsequently extracted from the sensing medium with the help of magnetic force. This was simultaneously accompanied by a real-time monitoring of the fluorescence change against a standard concentration. The study was validated using a comparative analysis with SPR to ensure reproducibility. The assays resulted in LODs of 0.26 U mL^−1^, 7.7 ng mL^−1^, and 0.55 ng mL^−1^ for CA-125, ApoA1, and β2-M, respectively. The sensor succeeded in distinguishing ovarian cancer patients from healthy individuals with 94% sensitivity and 98% specificity. Suaifan and Zourob developed electrochemical and optical-based biosensors for the analysis of PSA. They used carboxyl functionalised MNPs on a gold platform. The functionalised MNPs bonded with the N terminus of the PSA specific peptide and Au bonded with the thiol functionalities. Following proteolysis, an external magnetic field was applied to cleave the MNP-PSA peptide moieties from the Au platform. EIS revealed an LOD of 1 pg mL^−1^ with a detection range up to 1 μg mL^−1^. The optical analyses revealed relatively higher LODs of 100 pg mL^−1^ for SPR and 1 ng mL^−1^ for visual evaluation [166].

Lee et al. prepared the functionalised Fe_3_O_4_ core–Au shell structures on GSPE for the detection of eosinophil cationic protein (ECP), a biomarker for asthma [181]. The CYA labelled heparin (Hep) modified core-shell magnetic nanostructures amplified the electrochemical signal difference, thus improving the sensitivity of the biosensor. CV and SWV measurements revealed that the sensor can be used for a wide investigation range (1–1000 nM) with quite high sensitivity (LOD: 0.30 nM). Due to rising cases of myocardial infarction, various MNP-based SPR biosensors for cTnI have been reported in the last five years. In one such study, PDA wrapped Fe_3_O_4_ doped MWCNT (MMWCNT) was implemented to enrich cTnI dAb and used for magnetic extraction [182]. The sensing medium in this case was made up of a film of PDA and hollow gold nanoparticles (HGNP), which underwent self-assembly on a mercapto-functionalised gold platform prior to cAb immobilisation. The high surface area and the magnetic nature of MMWCNT improved target enrichment and allowed magnetic extraction. Wavelength modulated SPR provided responses from 1.25 ng mL^−1^ to 4 μg mL^−1^ of cTnI. Another study dispersed sodium oleate (NaOL) treated MNPs functionalised with cAb in water for the extraction of cTnI for further sensing on a gold nanorod (GNR)-based LSPR chip [183]. The NaOL treatment generated carboxyl groups on the MNP surface, encouraging cAb attachment. The fluorescent properties of Fe_3_O_4_ in addition to its high surface area contributed to an LOD of 2.5 ng mL^−1^.

The applications of MNPs are not restricted to merely electrochemical and optical sensors, but also include piezoelectric and magnetic sensors. Human α thrombin (HαT) is a biomarker for cardiovascular diseases and pulmonary metastasis. Sinha et al. reported HαT detection using a planar Hall magnetoresistive (PHR) sensor [184]. They used an Au film conjugated with thiolated DNA aptamer as the sensing platform and developed a sandwich immunoassay with biotinylated aptamer in the presence of the HαT analyte. Finally, streptavidin-coated MNPs were used to report a magnetic signal between 86 pM and 8.6 µM and a lower detection limit of 86 pM was achieved. Bayramoglu et al. reported a similar aptasensor for HαT based on piezoelectric transduction by a gold QCM [185]. Fe^+2^ and Fe^+3^ nanoparticles were suspended in poly(2-hydroxyethyl methacrylate-ethylene glycol dimethacrylate-vinylene carbonate) Mp(HEMAEGDMA-VC) microbeads and the microbeads were functionalised with thrombin binding aptamer (TBA). The QCM chip in this case was treated with Cys before TBA immobilisation to encourage glutaraldehyde coupling. The piezoelectric sensor sensor could quantify HαT in a linear range of 1.0–100 nM.

MNPs are also a popular choice for pathogen detection. Takemura and co-workers developed an LSPR-magnetofluoroimmunoassay (MFIA) for the ultrasensitive detection of norovirus (NoV), a pathogen responsible for infectious gastrointestinal disease, using a multifunctional nanocomposite of gold nanoparticles, magnetic nanoparticles, and CdSeS quantum dots, wherein anti-norovirus genogroup II antibody (antiNoV) conjugated the AuNP-MNP and the CdSeSQD [186]. The sensor could detect NoV-like particles (NoV-LP) in human faeces in a concentration range of 1.0 pg mL^−1^–5.0 ng mL^−1^ with an LOD of 0.48 pg nL^−1^. When tested for various types of clinical NoV, the LOD was 84 RNA copies mL^−1^. Jeong and Lim developed a magnetophoretic separation inductively coupled plasma mass spectrometry (InCP-MS) technique employing multicore magnetic nanoparticles (MMNPs) for the sensitive detection of *Salmonella typhimurium* (*S. typhi*), a pathogen responsible for gastroenteritis [187]. In this technique, MMNPs were doped with Cesium (Cs), silanised, and treated with (3-Aminopropyl)trimethoxysilane (APTMS). The capture probe in this study consisted of gadolinium-doped silica nanoparticles with antibody immobilisation (Gd-SilNP-cAb). The LOD of this sandwich-type assay was found to be 102 cells mL^−1^.

### 3.3. Gold Nanoparticles

Gold nanoparticles (AuNPs) are conductive materials, which possess a large surface area and exhibit unique optical properties. In AuNPs, a surface plasmon is confined, giving rise to LSPR. Therefore, their colour changes from red to yellow as their size increases from 100 nm [2]. AuNPs undergo oscillations, which are analogous to their metallic lattice. Depending on the shape of AuNPs, the heat and light scattering arising from the surface plasmonic decay may be affected. Hence, colour changes in nanospheres are less pronounced than GNRs. Another factor that can tune the optical properties of AuNPs is their degree of aggregation. This parameter can be harnessed for developing biosensors and optical immunoassays [133]. Multifunctional AuNPs are now being widely used for detecting various biomarkers for cancer [56,68,75,139,140,188], neurological disorders [31], diabetes mellitus [135,138], nucleic acids [17], amino-acids [70,99], hemoglobin [189], and a variety of pathogens [79,136,190,191].

Shan and co-workers developed a piezoelectric QCM aptasensor using silver enhanced AuNPs as labels for the determination of CCRF-CEM cells, which are T-cell biomarkers of acute lymphoblastic leukemia (ALL) [140]. The use of aminophenylboronic acid-functionalised AuNPs (APBA-AuNPs) resulted in signal amplification, providing linear responses in the concentration range of 2 × 10^3^–1 × 10^5^ cells mL^−1^ (LOD: 1160 cells mL^−1^). Yan et al. detected CD-10, another biomarker for ALL, using a label-free QCM-based sandwich immunosensor, relying on antibody immobilised glutathione functionalised AuNPs (Ab_2_-Glut-AuNPs) as signal amplification agents. The large surface area and superior conductivity of AuNPs allowed detection in the range of 1.0 × 10^−11^–1.0 × 10^−10^ M with a 2.4 × 10^−12^ M limit. 

Chaichi and Ehsani immobilised GOx on chitosan shells with Fe_3_O_4_ cores and integrated it with a luminol chemiluminescence system to develop an optical sensor for glucose [137]. They used gold nanoparticles to catalyse the luminol CL reaction as well as the reaction between GOx and glucose that lead to the generation of H_2_O_2_. The linear range of the neoteric sensor was 1 × 10^−4^–8.5 × 10^−7^ M and the detection limit was 4.3 × 10^−7^ M. Guo and co-workers reported a similar enzymatic sensor with an electrochemical detection mechanism [135]. Following the initial electrophoretic deposition of rhodium nanoparticles on a platinum electrode, AuNPs, Nafion and GOx were deposited. The sensor was reported to be selective against electroactive non-specific biomolecules, such as acetaminophen, UA, and AA, and had a lifespan of up to 90 days. The sensor provided a linear amperometric response from 0.05 to 15 mM with an LOD of 30 μM.

**Figure 9:**
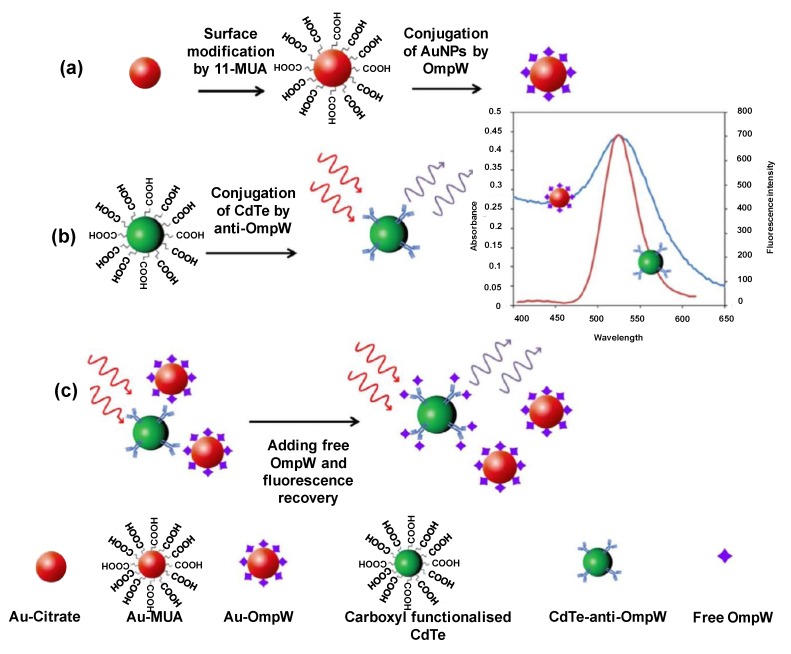
Schematic representation of (**a**) Au-OmpW synthesis, (**b**) carboxylated CdTe conjugation with OmpW antibody, (**c**) CdTe-FRET immunoassay [192].

AuNPs are also materials of choice for pathogen quantification. A FRET-based immunoassay used AuNPs for the competitive fluorescence quenching of CdTe quantum dots to detect outer membrane protein W (OmpW) of *Vibrio cholerae* [192]. The AuNPs were functionalised with 11-mercaptoundecanoic acid (11-MUA) and then conjugated with OmpW, as shown in Figure 9a. Figure 9b depicts the integration of carboxyl functionalised CdTeQDs with pAb of OmpW. When OmpW reacts with its antibody, the distance among the two nanoparticles reduces below 10 nm, causing energy transfer from CdTeQDs to AuNPs, thereby lowering the emission intensity. The fluorescence quenching increases with an elevation in the AuNP–OmpQ concentration by FRET. Competitive binding, as illustrated in Figure 9c, takes place when free OmpW is added to the system, ultimately leading to fluorescence recovery. The sensor allowed the quantification of OmpW in a linear range between 2 and 10 nM [192]. In another study, an enzyme-free electrochemical sensor was developed using a nanocomposite of Cu–Zr MOFs with aptamer as the signalling probe for the detection of *Pseudomonas aeruginosa* (*P. aeruginosa*) [193]. A highly conductive gold working electrode deposited with Super P, a stable form of carbon black, and AuNPs to obtain an enhanced and stable signal. This improved the electron transfer to adequate detection sensitivity. In addition to the exceptional conductivity, AuNPs played a key role in mAb immobilisation. CV, DPV, and EIS provided a detection range of 10–10^6^ cfu mL^−1^ and the LOD was found to be 2 cfu mL^−1^. Sabouri and co-workers established a chemiluminescence sandwich immunosensor for the detection of hepatitis B using functionalised AuNPs as capture probes [136]. The AuNPs were initially allowed to react with 11-MUA prior to conjugation with luminol and cAb. The detection probe consisted of dAb. The predominant ability exhibited by AuNPs to function as biological labels was harnessed to obtain a CL response between 0.12 and 30 ng mL^−1^ of Hepatitis B surface Antigen(HBsAg), with a limit of 14 pg mL^−1^. A study for Zika virus (ZKV) detection employed silsesquioxane polyelectrolyte (SiPy) as a stable support for AuNPs [194]. To construct the impedimetric biosensor, ssDNA was immobilised on an oxidised glassy carbon electrode (oxGCE) that was modified with AuNPs-SiPy. The sensor could measure the target in a wide concentration range (1.0 × 10^−12^–1.0 × 10^−6^ M) with a high sensitivity (LOD: 0.82 pM).

### 3.4. Silver Nanoparticles

Analogous to AuNPs, silver nanoparticles (AgNPs) are commonly used in medical diagnostics. The colour of AgNP solutions is also dependent on the particle size, owing to LSPR absorption. Similarly, their optical properties are a function of their degree of aggregation, shape, and size. However, the antimicrobial nature of AgNPs set them apart from AuNPs. In addition, they possess attractive electrical properties [2], with a more affordable cost than AuNPs. AgNPs are also utilised for enhancing the performance in a biosensing system. AgNPs have been widely used for SERS-based biosensors [133,140]. They demonstrate a higher extinction coefficient than AuNPs of the same size and undergo electrochemical oxidation more easily. Though the instability and functionalisation of AgNPs was considered challenging in the past, the synthesis methods and modification techniques have substantially improved them in recent years [195].

AgNPs have been used for the detection of various pharmaceutical and narcotic drugs, as well as for monitoring their effects on human. Raj and Goyal modified pyrolytic graphite (PyG) with a nanocomposite of ERGO and AgNPs to detect caffeine (CAF) and determine its effect on the concentration of estradiol, 1,3,5 (10)-estratrien-3,17 β-diol (EST) in women of child-bearing age [196]. EST is primarily a female growth hormone. AgNPs in conjugation with ERGO have a synergistic electrocatalytic effect arising from the storage capacity of electrons and the ability to supply electrons on demand. The voltammetric sensor provided wide detection ranges of 0.001–200 µM and 0.001–175 µM and LODs of 0.54 nM and 0.046 nM for EST and CAF, respectively. The sensor was highly selective against AA, UA, xanthine, and hypoxanthine [196]. Mao et al. developed a SERS sensor for the determination of methylamphetamine (MAMP), a popular illicit drug, using Au_shell_-Ag_core_ nanoparticles [197]. The SERS performance of the shell–core structures was much more pronounced in comparison to AuNPs. The sensor quantified MAMP with the help of MAMP aptamer in the range of 0.5–40 ppb, and the detection limit was found to be 0.16 ppb. Bagheri and co-workers developed a patulin sensor with the help of AgNP–ZnMOF capped with a molecularly imprinted copolymer of 3-Aminopropyl)triethoxysilane (APTES) and tetraethyl orthosilicate (TEOS) [198]. AgNPs displayed a mimetic activity in the ZnMOF, thereby increasing the number of active sites for the H_2_O_2_–terephthalic acid reaction, resulting in high fluorescence. The sensor provided a linear response between 0.1 and 10 µM, and the detection limit was 0.06 µM.

Zheng and co-workers reported a highly sensitive and simultaneous sensing of multiple breast cancer biomarkers by developing a SERS microfluidic chip sensor [199]. The AgNPs were functionalised with 5,5’-dithiobis-(2-nitrobenzoic acid) (DTNB) and subsequently modified with 4 mercaptobenzoic acid (4MBA) and antibodies as Raman reporters. The unreacted sites were capped with bovine serum albumin (BSA). Antibodies were immobilised on a SERS substrate and a sandwich immunoassay provided a linear detection response for cancer antigens CA 153, CA 125, and CEA in the respective ranges of 0.001 U mL^−1^–1 kU mL^−1^, 0.001 U mL^−1^–1 kU mL^−1^, and 0.1 pg mL^−1^–100 ng mL^−1^ in serum. The LODs were 0.01 U mL^−1^, 0.01 U mL^−1^, and 1 pg mL^−1^, respectively. In another study for PSA detection, a nanomaterial–conductive polymer nanocomposite, graphene-poly(3-aminobenzoic acid) (GP-P3ABA), was used for electrode modification and porous-hollowed-silver-gold core–shell nanoparticles (PHAu_shell_-Ag_core_NPs) amplified the signals by acting as labels in the system [200]. A 3-fold current response was obtained for PHAg_core_-Au_shell_NPs against pure AuNPs. A detection limit of 0.13 pg mL^−1^ with a detection range of 0.01–80 ng mL^−1^ was observed. Xia et al. reported a peptide-based aptasensor for human chorionic gonadotropin (HCG), a well-known biomarker for many kinds of cancer, using AgNPs as a redox reporter species [201]. The study converted an AgNP-based colorimetric assay into electrochemical analysis. The binding of HCG on the Au electrode induced the peptide probe to be deprived of its capability to trigger the on-site formation of AgNPs network architecture on the surface of the electrode, resulting in a highly attenuated LSV response. Consequently, an LOD of 0.4 mIU mL^−1^ was reported.

Yang et al. developed an ultrasensitive electrochemiluminescent biosensor for cholesterol monitoring by exploiting the exceptional catalytic activity of AgNP–BSA–MnO_2_ nanosheets [202]. Cholesterol oxidase (ChsOx) was immobilised on the nanosheets. Efficient signal amplification in the luminol–H_2_O_2_ CL system was observed and the linear detection response was obtained for cholesterol concentration between 0.21 and 1667 μM, with a detection limit of 0.07 μM. Another study reported a rapid, multifunctional, specific, and highly sensitive electrochemical platform to sense, eliminate, and inactivate *Staphylococcus aureus* (*S. aureus*) using vancomycin (Van) immobilised silver nanoparticle–three-dimensional zinc oxide nanorod arrays AgNPs/3D-ZnO on a fluorine doped tin oxide electrode (FTO) [203]. The AgNPs were particularly chosen for their anti-microbial nature. The impedimetric sensor displayed a detection range of 10^3^–10^7^ cfu mL^−1^ with an LOD of 330 cfu mL^−1^.

The exceptional electrical conductivity possessed by inorganic materials like AgNPs makes them ideal candidates for biosensors relying on electrochemical detection, as listed in Table 4.

### 3.5. Nanocages, Nanoshells and Nanowires

**Figure 10:**
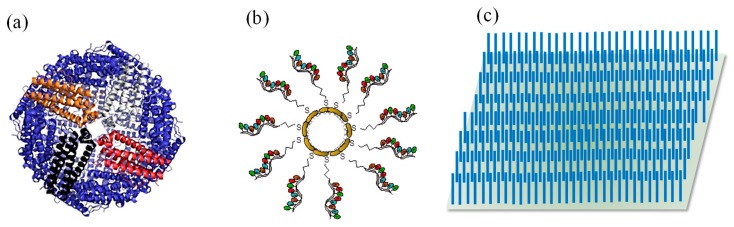
(**a**) Nanocage [217], (**b**) nanoshells [218], (**c**) nanowire arrays.

Nanocages (NCgs) constitute a new class of nanomaterials made up of noble metals with a hollow interior and porous walls (Figure 10a). Their size generally ranges from 10 to 150 nm. Since these nanoparticles are cube-shaped, their optical properties differ from spherical nanoparticles, allowing them to absorb light in the NIR region of electromagnetic spectrum. Depending on the extent of precursor added to the system, their degree of LSPR can be tuned. NCgs are used in biosensing due to their ability to absorb and scatter NIR light [2]. Mei et al. supported palladium copper nanocages (PdCuNCgs) on RGO for CAT determination [153]. RGO prevents the agglomeration of the Pdu units during repeated catalysis. The RGO–PdCuNCgs acted as an immobilisation platform for laccase enzyme. Due to the large surface area and high conductivity of PdCuNCgs, the electrochemical sensor was able to detect 5 μM–5.155 mM CAT with an LOD of 1.5 μM. Zhao and co-workers reported a facile fabrication of a peroxide mimetic glucose biosensor using iron nanoparticle loaded Cu_3_O_4_ hollow nanocage (FeNPs@Co_3_O_4_ HNCgs) [215]. The nanocages displayed 195 times higher affinity for H_2_O_2_ in comparison to HRP. The sensor detected glucose in the 0.5–30 μM concentration range and the detection limit was 0.05 μM. Another study loaded GQDs on surface villous AuNCgs to develop an electrochemiluminescence device based on Au nanoflower functionalised paper working electrode (AuNFl-PWE) for the detection of CA-153 on the surface of MCF-7 cells [216]. The sandwich assay constituted primary antibodies immobilised on AuNFl-PWE as the sensing platform and the secondary antibody functionalised GQD-AuNCgs as signal probes. The surface structure of the NCgs facilitated electron transport and allowed a higher number of GQDs to be loaded. The rapid and low-cost device provided linear responses between 0.005 and 500 U mL^−1^ with a good sensitivity (LOD: 0.0014 U mL^−1^).

Nanoshells (NShs) are spherical nanoparticles which generally consist of a thin metallic outer shell with a dielectric core, as illustrated in Figure 10b [217]. NShs make ideal components for optical sensors, as their quantum plasma oscillation feature can be easily adjusted by manipulating the composition and size of the core and shell [218]. It is feasible to functionalise biomolecules, such as proteins, on these moieties to tailor the inertness or bioreactivity of nanoshells, making them an ideal choice for therapeutic applications and biosensing [219,220]. Yang et al. used hollow porous nanoshells (HPNShs) of a PtAg bimetallic alloy modified on a GCE to fabricate an ultrasensitive electrochemical biosensor for superoxide anion (O_2_^•−^), a well-known regulatory mediator in immune and signal processes [221]. The porous surface and the interconnected grains of the nanoshell make it especially conducive to electrochemical sensing. The hollow structure in association with the porous surface promoted greater reaction medium contact. The copious pores integrated with the interconnected backbone assisted the unlimited mass and electron transport during the electrochemical catalysis [222,223]. The biosensor provided an exceptional response for real time O_2_^•−^ sensing in cellular medium, offering a detection range of 0.8–1080 nM and a detection limit of 0.2 nM. Phan and co-workers used Cu_shell_-Au_core_ nano particles for the determination of cultural filtrate protein (CFP-10), a *Mycobacterium tuberculosis* antigen, with the help of a dot-blot immunoassay, which allowed a highly sensitive detection by the naked eye [149]. Prior to growing the Cu nanoshells, gold binding polypeptide antibodies (GBPAb) were immobilised on the AuNPs. The CuNShs induced the appearance of colour intensity in the 0.015–1 ng mL^−1^ concentration range and the LOD was 7.6 pg mL^−1^. Due to the ease of evaluation using the naked eye or a smart phone camera, the sensor holds great promise for POC testing. Gao et al. used an in situ amplified colorimetric immunosensor based on an extremely efficient peroxidase mimetic system using Pt_shell_-Au_core_ urchin-like nanohybrids (Pt_shell_-Au_core_NHs) [224]. The urchin-like nanhohybrids could vastly outperform HRP in performance and provided linear responses from 5 to 500 pg mL^−1^ with an LOD of 2.9 pg mL^−1^.

Nanowires (NW) belong to the class of monodimensional nanoparticles, which also includes nanotubes, nanobelts, and nanorods (Figure 10c). The length of NWs is at least 1000 times their diameter. Semiconducting substances such as Si, InP, and GaN, as well as dielectric materials such as TiO_2_ and SiO_2_, can be used to make NWs [2]. Noble metal NWs exhibit thickness dependent LSPR properties. SiO_2_ NWs find often implementation in FET biosensors, owing to their conductive properties, and hold enormous potential for healthcare biosensing. Signal transduction based on NWs acting as substrates for receptor immobilisation to allow binding with various biomolecules results in sensitive detection, rapid analysis, and a scope for miniaturisation [155]. A silicon nanowire (SiNW)-based microfluidic electrical sensor was recently reported by Nuzaihan and co-workers for the sensitive recognition of the dengue virus (DENV) DNA oligomer [156]. The SiNW was synthesised by a top-down approach on a silicon-on-insulator (SOI) wafer. The SiNW was then functionalised by surface modification, DNA immobilization, and DNA hybridisation. This neoteric molecular gate control mechanism allowed the sensor to achieve an LOD of 2.0 fM. Kim et al. developed a silicon nanowire FET biosensor using a honeycomb nanowire (HCSiNW) architecture for the ultrasensitive detection of cTnI [225]. The device showed exceptional sensitivity and selectivity due to the Debye effect. Antibodies of cTnI were immobilised on the HCSiNW surface. The LOD of this sensor was 5 pg mL^−1^ and the detection range was 5 pg mL^−1^–5 ng mL^−1^. In another study, a nanohybrid of three-dimensional platinum nanowire array (PtNWA) and AuNPs was used to develop a sensitive enzyme-based amperometric glucose sensor [226]. GOx was then immobilised on the nanohybrid. The vertically aligned platinum nanowires had a greater density of AuNPs in comparison to a 2D planar modification. The nanowires were responsible for improving the signal-to-noise ratio by enhancing the electron density on the electrode surface. CV and amperometric measurements proved that the sensor achieved a detection limit of 15 μM with a detection range of 15 μM–2.5 mM.

## 4. Organic Nanomaterial Applications for Healthcare Biosensing

Barring an exception to some of the ultramodern molecular machines, most of the organic nanomaterials are polymeric in nature. Interest in polymeric nanomaterials for biomedical applications such as drug delivery and medical diagnostics has escalated. This can be attributed to their biocompatibility, inherent inertness, and flexibility in design. Such nanomaterials are thermally stable and relatively inexpensive to produce. Nanostructured films involve facile preparation procedures, are easy to handle, and may even be recovered after use [227]. Molecularly imprinted polymeric nanoparticles (nanoMIPs), which are cross-linked polymers that can selectively conjugate to a desired biomolecule, hold promise as biomimetic substitutes to antibody receptors [228]. Dendrimers constitute another class that has captivated researchers in the area of clinical diagnostics. The star-shaped hyperbranched structures allow scientists to tune the properties of various biomolecules. The functionality, size, and shape of dendrimers can be manipulated by making changes to their exterior surface, interior dendritic structure, or the central core [3]. Nanostructured hydrogels, which are 3D polymeric networks made up of cross-linked polymer chains, are able to alter their structure and volume in response to changes in pH, chemical environment, temperature, light, or magnetic or electric field, thereby being termed as stimuli-responsive smart nanomaterials [2]. Hyperbranched polymeric nanoparticles are structurally different from dendrimers, since they possess linear units in addition to dendritic and terminal units. They possess advanced optical, electrical, and magnetic properties, making them supreme elements in various kinds of biosensing systems [229]. Polymeric nanocomposites are ideal transducers because of their morphological variations, elegant responsibility, and simple synthesis procedures. They are low in cost and offer high signal-to-noise ratios and can be employed in sensors based on DNA, aptamer, or antibodies [230].

Molecular machines are miniaturised devices that are propelled in the liquid medium either by themselves or by external acoustic, electric/magnetic, or catalytic energy sources [231,232]. Some such nanomotors are Janus particles, which exhibit different properties on different parts of the material. They can be functionalised with molecularly imprinted polymers, as well as biomolecules such as oligodeoxynucleotides (ODN), to function as receptors [231].

### 4.1. Nanostructured Films

To fabricate sensors for detecting various parameters in a biological environment, it is essential for all the biosensor components to be biocompatible. The materials should be nontoxic and recalcitrant to bodily fluids and tissues. Concomitantly, it should also not prompt chronic or acute response from tissues or cause inflammation. To this end, nanolayers or polymeric nanofilms provide an efficacious solution due to their protective nature, in addition to being used as sensing mediums wherein the refractive index of a nanolayer is changed due to an alteration in the quantity of the measurement variable [3].

Nanostructured films can act as an ideal transducing element capable of transferring the stimuli from the analyte as well as facilitating covalent functionalisation. Exploiting this virtue of nanofilms, Rahmanian and Mozaffari deposited ZnO–poly(vinyl alcohol) hybrid films on an FTO electrode to develop an enzymatic urea biosensor. The impedimetric biosensor utilised the hybrid films as transducers and harnessed the hydroxyl groups from polyvinyl alcohol (PVA) for the covalent immobilisation of the urease enzyme. The electrostatic repulsion from the nanofilm hindered the binding of anionic interferents. CV and EIS measurements provided a detection limit of 3.0 mg dL^−1^ with a linear response between 5.0 and 125.0 mg dL^−1^ [233]. Enzymes and, generally, proteins are important structured elements that have found applications in biosensing [234]. Another class of promising sensing elements is based on carbohydrates or glycopolymers [235]. A representative example of nanostructured films as an impedimetric element for bio-recognition was reported by Kraatz [236]. Forming a film of transmembrane glycoproteins called toll-like receptors (TLR) on a flat conductive surface results in effective sensors that recognize molecular patterns or signatures of pathogens. TLR specifically reacts with lipopolysaccharide (LPS) endotoxin, which is a glycolipid located at the outer membrane of Gram-negative bacteria. Applying EIS, the limit of detections found for *E. coli* and *Salmonella* were 1.3 × 10^−4^ and 1.5 × 10^−4^ EU mL^−1^, respectively. Pedro et al. were the first to develop intrinsically conducting polymer (ICP) nanofilms as fluorescence quenchers for DNA sensing [227]. They performed a comparative study between PPy and polyaniline (PAni) nanofilms on a polyethylene terephthalate (PET) substrate. The biomarker was 6-carboxyfluorescein-labeled single-stranded DNA (FAM-ssDNA) from *Leishmania infantum* (*L. infantum*) parasite. The thickness and hydrophobicity of the films resulted in a high signal-to-noise ratio. The detection limit for PAni/PET was 1.3 nM, whereas for PPy/PET it was 1.1 nM. Another similar study using electrodeposited ICP utilised CV to deposit a Nafion nanostructured poly(aniline) (PAni) film for the ultrasensitive detection of creatinine(CRN), a common biomarker for nephrological diseases [237]. The film promoted electron transfer and acted as a substrate for creatinine deiminase (CRND) immobilisation. The amperometric sensor achieved a detection range of 0.005–0.4 mM and the LOD was 0.005 mM. 

Phetsang’s group fabricated an amperometric biosensor using nanocomposite film of Pt, RGO, and P3ABA deposited on an screen printed carbon electrode (SPCE) for the enzymatic detection of glucose and cholesterol [238]. The nanofilm modified electrode showed exceptional electrocatalytic oxidation for H_2_O_2_ and the LOD and detection ranges for glucose were 44.3 μM and 0.25–6.00 mM, respectively. The detection limit for cholesterol was found to be 40.5 μM within a detection range of 0.25–4.00 mM. Another study with a much lower cholesterol LOD fabricated a similar nanofilm-based cholesterol sensor using PDA nanofilm structured with PB and oxidised MWCNTs [239]. The PDA film promoted ChsOx immobilisation. The sensor showed a high affinity for cholesterol. The sensor was functional in the 4–400 μM concentration range with a 1.5 μM limit. Liu and co-workers reported an ultrasensitive electrochemical glucose biosensor using Prussian blue nanocube (PBNC) films [240]. Graphene was used to promote the conjugation of GOx with PBNC. The PBNC film acted as an “artificial peroxidase”. An LOD of 10 μM with a detection range of 0.001–0.8 nM was achieved.

### 4.2. Nanostructured Hydrogels

Nanostructured hydrogels (NHg), or nanogels, are nanoscale 3-D cross-linked hydrophilic polymeric networks that can alter their chemical structure and volume with the help of stimuli from the external environment. Hence, they are termed stimuli-responsive smart materials. Furthermore, they have a large surface area withal swelling translation, biocompatibility, and stability [241]. Based on the function of nanogels in a biosensing system, their applications can be categorised into: (i) encapsulation vehicles; (ii) multifunctional stimuli-responsive materials; and (iii) sensory membranes [242]. 

Nanostructured hydrogels, owing to their large surface area, are excellent for encapsulating fluorescent molecules, facilitating optical biosensing. Based on this approach, Guo’s group developed a two-photon fluorescent sensor for the detection of cysteine (Cys) [243]. The “turn-on” probe comprised of triarylboron luminogen 4-(dimesitylboryl)-*N*,*N*-diphenylaniline with π conjugation throughout and a methylene spacer attached to a maleimide moiety (DMDP-M). A nonogel of the triblock copolymer poloxamer 407 (POX407) was employed to encapsulate the hydrophobic DMDP-M fluorescent probes. In the presence of Cys, photoelectron transfer in the probe was hindered, generating fluorescence. The system presented a low LOD of 0.18 μM. Due to its high cell-membrane permeability and biocompatibility, the system also found successful application in NIH/3T3 cell line. Cao et al. developed a similar sensor for intracellular pH (pHi) in cytosol using a polyurethane (PUR) nanogel loaded with 8-hydroxypyrene-1-carbaldehyde (HPC), a fluorescent dye [244]. The dual-emissive ratiometric detector allowed in vivo sensing in the pH range of 4–10 by undergoing a reversible and large hypsochromic shift of 100 nm in the red–green region of the visible spectrum.

**Figure 11:**
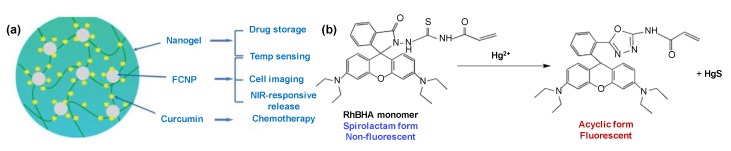
(**a**) Multi-functional nanogel capable of drug storage, temperature sensing, cell imaging, and drug delivery [245], (**b**) Ring opening reaction of rhodamine B derivative induced by Hg^2+^ ions [246].

Multi-functional stimuli-responsive NHgs change their volume dramatically due to a change in the environmental stimuli. Exploiting this virtue, Wang et al. developed a photoluminescent sensor using NHgs for optical temperature sensing and NIR responsive drug release [245]. Temperature detection in the range of 16–50 °C was possible by loading a poly(*N*-isopropylacrylamide-co-acrylamide) (pNIPAm-Am) nanogel loaded with fluorescent carbon nanoparticles (FCNPs) (Figure 11a). Environmental temperature sensing was accomplished by manipulating the photoluminescence intensity, which was possible due to the reversible thermo-responsive transition between swelling and shrinking of the nanogel, which altered the physicochemical environment around the FCNPs. Such NHgs are called mingle structured multifunctional stimuli responsive nanogels. An alternative to this architecture is the incorporation of various kinds of nanoparticles within a nanogel shell. Li and co-workers produced such a structure using NaYF_4_:Yb^3+^–Er^3+^ nanocrystals as the core and a thermo-responsive nanoshell comprising of a free radically polymerised *N*-isopropylacrylamide and *N*-acrylyl-*N*′-rhodamine B acylhydrazine thiourea nanogel (pNIPAm-RhBHA) [246]. In the presence of 0.0–0.099 mM of Hg^2+^, the rhodamine moieties were transformed to 1,3,4-oxadiazoles, causing fluorescence, as illustrated in Figure 11b. The system also responded to pH, metal ions, and temperature. Kim and Li reported a similar sensor using poly(*N*-isopropylacrylamide) (pNIPAm) nanogel and fluorescein (Fsc) shells with vinyl functionalised carbon dot (ViCD) cores capable of detecting temperature over a wider range in comparison to the previous study [247]. The ratiometric sensor emitted reversible fluorescence from 25 °C to 45 °C because of the swelling and shrinking of the hydrogel in response to temperature. The main reason for these thermo-responsive volumetric changes can be attributed to a perturbation in the intermolecular hydrogen bonds between pNIPAm and water in the aqueous solution that takes place due to phase transition above a lower critical solution temperature.

The sensitivity, accuracy, and durability of biosensors are greatly affected by its membrane and pose a significant challenge. Sun et al. reported a chitosan–pNIPAm nanogel membrane for sensing temperature and ethanol on microchips [248]. The membrane enabled volume shifts and provided a platform of nanovalves in the channels of the chip. This strategy holds promise for in situ smart nanomembrane fabrication for fabricating lab-on-a-chip type sensors, detectors, and controlled release systems. Besides their function as membranes, NHgs may function as other elements in a biosensing system. An interesting application of such a system is the glucose sensitive artificial muscle developed by Lee and co-workers [249]. They harnessed the glucose sensitive nature of boronic acid (BoA) by conjugating it with a nanogel of HyA and cholesterol. This nanocomposite was subsequently used for MWCNT yarn actuation due to their reversible swelling/shrinking. Zhao et al. exploited the negative charge and poor conduction of poly(acrylic acid) (pAAc) to impede electron transfer and repel the [Fe(CN)_6_]^3−/4−^ mediator [250]. They used the nanogel as an amplification agent to develop an ultrasensitive impedimetric aptasensor for CEA. The sensor provided a linear impedimetric response in serum from 10.0 fg mL^−1^ to 10.0 ng mL^−1^ and the LOD was 1.4 fg mL^−1^.

### 4.3. Dendrimers

**Figure 12:**
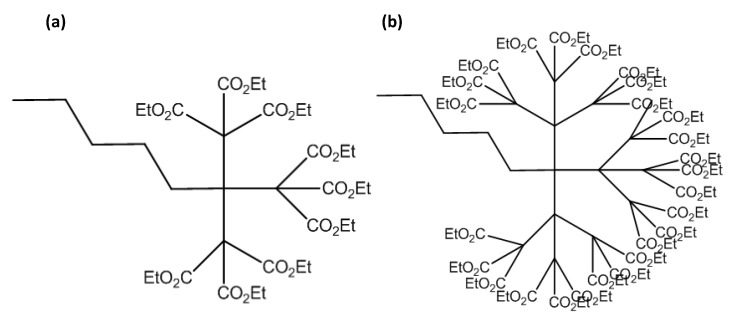
(**a**) First-generation (G1) dendrimer, (**b**) second-generation (G2) dendrimer.

Dendrimers are molecular nanoparticles with a covalent assembly of atoms distinguished into three architectural components [2]: (i) a central core of initiator or metallic ions; (ii) generations representing interior layers of repeating units bound to the core (Figure 12); (iii) terminal functionality representing the exterior of the particle which is attached to the outermost generation of the interior layers.

The multifunctional interface in addition to their globular ship makes dendrimers potential candidates for fabricating hybrid, multilayer nanomaterials. One of their most interesting virtues is the ability to self-assemble into superstructures at material peripheries. Their unique structural features, such as hyperbranching, spheroidal surface, nanoscopic size, and voluminous interior, in addition to high solubility, fluidity, and reactivity, have made dendrimers one of the newest classes of macromolecular sensing devices for medical diagnosis [3].

Although analytes such as proteins and dendrimers readily attach to inorganic biosensor surfaces, they may lose their activity due to denaturation. An interesting strategy to avoid this phenomenon is the immobilisation of dendrimers on the biosensor surface [251]. A similar strategy for the immobilisation of streptavidin on PAMAM functionalised GCE was performed using electrostatic interactions upon drop coating of the electrode in the study of Soda and Arotiba [252]. An electrochemical interrogation of the GCE-G3(PAMAM)-Str proof-of-concept DNA biosensor was performed in phosphate buffer, H_2_O_2_, and ferrocyanide. The stable, electroactive supramolecular architecture of the dendrimer–streptavidin platform acted as a versatile substrate for the immobilisation of any biotinylated receptors. Erdem et al. functionalised MNPs with G4 PAMAM dendrimers to achieve improved voltammetric sensing of DNA oligonucleotide (DNA-ODN) upon electrode response [253]. G4 PAMAM with a core of 1,4–diaminobutane was used to functionalise the streptavidin coated MNPs. The genomagnetic assay obtained by voltammetric detection had an LOD of 4.09 μg mL^−1^.

Hao et al. developed a microfluidic *E. coli* O157:H7 sensor using a combination of aptamers and dendrimers [254]. They established the superiority of seventh-generation (G7) PAMAM dendrimers over G4. The aptamer–dendrimer modification on the plasma-treated poly(dimethyl siloxane) (PDMS) surface furnished multiple binding sites to boost target capture at high throughput rates. The efficacy of the sensor was evaluated with fluorescent measurements and a low LOD of 10^2^ cells mL^−1^ with a wide linear response of 10^2^–10^7^ cells mL^−1^ was obtained. Elencheziyan and Senthilkumar encapsulated G3 PAMAM dendrimers with haemoglobin (Hbg) immobilised AuNPs for sensing H_2_O_2_ electrochemically [255]. CV and amperometric responses provided a linear response for the concentrations between 20 and 950 µM with 6.1 µM as the limit.

Shukla et al. reported an electrochemical urea biosensor consisting of zirconia (ZrO_2_) and G2 poly(propylene imine) (PPI) dendrimers co-deposited on an SPCE with the help of CV [256]. The amperometric sensor could determine urea in the range of 0.01–2.99 mM. The G2(PPI) acted as a biocompatible nanolayer encapsulating ZrO_2_ and played the role of a covalent attachment site. Borisova’s group developed a new nanohybrid using G4 PAMAM with magnetic PDA nanoparticles decorated with platinum nanoparticles (PtNPs) [257]. The nanohybrid was assembled on a GCE covered with GO-carboxymethyl cellulose (CMC) on a layer-by-layer basis to obtain a nanostructured electrode, which was used to immobilise xanthine oxidase (XOx) for fabricating a biosensor for the determination of xanthine, an alkaloid bronchodilator used to treat asthma symptoms. The sensor achieved a broad detection range of 50 nM–12 μM and the LOD was 13 nM. Dervisevic’s group developed a neoteric amperometric biosensor for urea, relying on the self-assembled immobilisation of urease on CYA-modified PAMAM grafted MWCNT [258]. They performed experiments using first-generation (G1) to G5 PAMAM dendrimers and concluded that G5 PAMAM presented an exceptional performance with a rapid detection time of just 3 s, wide detection range of 1–20 mM, and an LOD of 0.4 mM.

### 4.4. Hyperbranched Polymeric Nanoparticles

Hyperbranched polymers (HBPs) are structurally similar to dendrimers but there are certain key distinctions. In addition to the dendritic cores and terminal units present in dendrimers, HBPs also contain linear units. Furthermore, the dendritic and linear units are distributed arbitrarily throughout the macromolecular architecture, giving rise to irregularities in the structure. As opposed to dendrimers, HBP synthesis generally involves a single step reaction [229]. Due to low chain entanglements, linearity, and a large number of functional groups, HBPs present interesting thermal, rheological, electrochemical, and luminescent properties, presenting enormous scope in different kinds of biosensing systems [259].

Niu’s group developed a hyperbranched polyester to mimic heparin for thrombin detection [260]. The sulfonated HBP was functionalised on a GCE with the help of mercaptopropyltrimethoxysilane (MPTMS) and AuNPs prior to thrombin binding aptamer (TBA) immobilisation. The label-free aptasensor was also used for in vitro studies to develop hemolysis assays, platelet, and whole blood adhesion tests as well as to understand the changes in the morphology of red blood cells. Exceptional sensitivity was observed with an extremely wide detection range of 2.7 pM–27.0 μM and a limit of 31 fM. In another study, human sirtuin 1 (SirT1), a molecule which has potential anti-aging properties, was successfully detected using cross-linked hyperbranched azo polymer (HAP) [261]. Initially, indium tin oxide (ITO) was coated with PDA and then dipped in an HAP–AuNP colloidal solution. Finally, the electrode was coated with cAb and BSA was used to block the free sites. A sandwich immunoassay was obtained using dAb in conjugation with a secondary antibody Ab_2_ immobilised on a TiO_2_–AuNP nanocomposite. DPV provided linear electrochemical responses between 1.0 and 500 ng mL^−1^ and the LOD was 0.28 ng mL^−1^. A similar ultrasensitive CEA sandwich immunosensor using hyperbranched polyester for electrochemical detection was reported by Miao’s co-workers [262]. This study was conducted using an ITO electrode, wherein hyperbranched 2,2-bis(hydroxymethyl)propionic acid DMPA was grafted to the surface modified to obtain carboxyl functionalised end groups. Subsequently, a cAb was immobilised on the electrode. An HRP-labelled AuNP with dAb was used as a signalling probe. A detection range between 0.01 and 80 ng mL^−1^ was obtained with an LOD of 2.36 pg mL^−1^.

It is important to maintain adequate Cu^2+^ levels in the body, as its deficiency can result in bone abnormalities and neutropenia, whereas its excess can cause damage to kidneys and the liver and may even prove fatal. Therefore, Wang et al. developed an optical sensor for Cu^2+^ detection using hyperbranched polyethyleneimine (PEI) modified with formaldehyde and capped with AuNPs [263]. Detection was possible by visual evaluation as well as fluorescence and UV-visible spectroscopy techniques. The linear response at pH 4.0 was obtained in the 0.15–23 µM concentration range and the LOD was 77 nM. Romero and co-workers exploited the self-assembly of hyperbranched 2,2-bis(methylol)propionic acid (bis-MPA) on the surface of MWCNT to a fabricate an amperometric sandwich-type lactate sensor [264]. A platinum electrode functionalised with the aforementioned 3D scaffolds was used as a matrix to wire lactate oxidase (LOx). The HBP(bis-MPA) reduced the elastic characteristics of the enzyme matrix, thereby bringing an increment in the diffusion coefficient. This resulted in a lower detection time due to in situ regeneration of the enzyme mediator (O_2_). The linear detection range was 0.9 µM–1.5 mM and the limit was 0.9 µM.

Sedki’s group developed a nanocomposite of reduced graphene oxide (RGO) with hyperbranched chitosan to develop sensor platform for monitoring cell viability [265]. The target bacterium was *P.* *aeruginosa* and its viability was tested in the presence of various antibiotics, such as ciprofloxacin, simvastatin, and kanamycin. The OD_600_ LOD was 0.025 and the linear response range was between 0.025 and 0.5. Niu and co-workers reported an immunosensor for the label-free detection of α fetoprotein (AFP) using hyperbranched polyester with nitrite end groups in conjugation with a chitosan–AuNP functionalised GCE [266]. The modified electrode was then used as a surface for AFP antibody immobilisation and the free sites on the surface were blocked with BSA. The sensor displayed a linear response from 0.1 to 120 ng mL^−1^ of AFP and a very low LOD of 55 pg mL^−1^ was observed. In one study, hyperbranched ferrocene polymer was used for the detection of dihydronicotinamide adenine dinucleotide (NADH) in association with PtNPs. Polymethyldiundecenylsilane (PMDUS) and polydiallylmethylsilane (PDAMS) were the hyperbranched polymers chosen to interact with ferrocene. Both the respective assemblies had a synergistic effect with PtNPs on the catalysis of the sensing and exhibited wider detection ranges in comparison to sensors without PtNPs. The LOD for PDAMS assembly was 4.78 μM, which was lower than the 6.18 μM limit observed in the case of PMDUS.

### 4.5. Covalent Organic Frameworks

Covalent organic frameworks (COFs) consist of porous polymers linked to each other via covalent bonds in a crystalline assembly. They are capable of integrating organic blocks in an atomically accurate ordered structure with the help of strong covalent linkages [267]. It is possible to control the structure and size of the pores in a COF and such frameworks show high surface area, thermal stability, and permanent porosity [268,269]. The scope of COFs as components for biosensing was proposed in 2014 [270], wherein amino functionalisation allowed the adsorption of DNA and proteins in the framework. COF-based sensors show promising prospects in the detection of various antibiotics. As COFs are rich in N sites and show high porosity, it is possible to enhance their fluorescence quenching efficacy with the help of adsorption-triggered pre-concentration and rigidifying-induced fluorescence improvement. Tang et al. introduced aggregation-induced emission luminogens such as diphenamidine hydrochloride in a covalent traizine framework nanosheet for the sensitive and highly selective detection of nitrofurans, such as nitrofurazone (LOD: 4.97 ppb), nitrofurantoin (LOD: 8.08 ppb), and furazolidone (LOD: 13.35 ppb) [271]. The limit of detection reduced nearly four-fold when the luminogen was replaced with phenylamidine hydrochloride. In another study, COFs were used as amplifying agents in a nanocomposite with graphene oxide [272]. The nanocomposite was used in conjunction with molecularly imprinted polypyrrole and GCE to generate an electrochemical sensor for the determination of sulfadiazine and acetaminophen. The sensor showed a performance identical to that of an independent high-performance liquid chromatography assay [272]. Recently, Wang and co-workers developed a COF with imine bonds generated by the polycondensation of melamine and 1,3,6,8-tetrakis(4-formylphenyl)pyrene [273]. The COF showed an extensive specific surface area (495.5 m^2^ g^−1^), large porous cavities, and a structure resembling nanosheets with abundant ene, carbonyl, imine, and amine functional groups, along with a high charge carrier mobility which facilitated the immobilization of DNA aptamers. This COF-based aptasensor yielded extremely low LODs for the detection of enrofloxacin (6.07 fg mL^−1^) and ampicillin (0.01 fg mL^−1^) [273].

In addition to antibiotics, COFs are useful for the detection of several disease biomarkers, particularly those indicative of cancer. As a platform, they show high versatility among various detection systems. Therefore, Ai and He developed a chromogenic visual colorimetric system for sensing 3,3′-diaminobenzidine, which is suspected of carcinogenic and mutagenic properties [274]. The sensor utilized the large π-conjugation system present in imine-linked COFs. The high energy electrons enhanced the extinction coefficient of the system and allowed sensitive and selective visual detection down to 5 μM with an LOD of 900 nM in UV-vis assisted colorimetric assay [274]. Similarly, Yan et al. developed an electrochemical sensor for the detection of Michigan cancer foundation cell line (MCF-7) as well as human epidermal growth factor receptor (hEGFR) [275]. The 2D porphyrin-based COF was rich in nitrogen-bearing moieties with large cavities and an excellent aqueous stability, low toxicity, and high bioaffinity encouraged the immobilization of aptamer strands. Electrochemical detection with differential pulse voltammetry and electrochemical impedance spectroscopy allowed low LODs for both the targets with high reproducibility and recyclability [275]. Liang’s group developed a similar electrochemical sensor using magnetic COFs for the determination of PSA in buffer as well as serum [276]. The sandwich immunosensor consisted of an AuNP–phosphorene nanocomposite platform for the immobilization of primary antibodies. Secondary antibody functionalised AuNP loaded magnetic COFs along with methylene blue were responsible for electrochemical signal generation. The research presented an effective assay for PSA detection in the range of 100 fg mL^−1^ to 10 ng mL^−1^, utilising an optimum combination of the superior electron transfer of phosphorene, effective enrichment of methylene blue in the COF, and the excellent catalytic activity of Fe_3_O_4_ molecules [276].

Two-dimensional COF layers often exhibit a great amount of attraction, preventing the formation of stable thin films. It is also possible to generate COFs from three-dimensional kenaf stem-derived microporous carbon by soaking it in a reactive liquid [277]. Yang et al. used this technique to generate COFs, which were subsequently integrated with electrodes to fabricate electrochemical sensors [278]. Copper nanoparticle deposition on these COFs enabled glucose detection, whereas platinum nanoparticle-deposited COFs allowed the determination of H_2_O_2_. A similar metal nanoparticle doped COF was proposed by Gu’s group for the detection of aflatoxin B1 [279]. In this work, AuNPs were embedded in COF, which was further functionalised with molecularly imprinted poly(*o*–aminothiophenol). The piezoelectric sensor showed linear responses in a wide concentration range (50 pg mL^−1^–75 ng mL^−1^) with significant recoveries (87.0–101.7%) in real samples [279]. In another study, AuNP-doped COFs were used for sensing cardiac troponin I (cTnI) [280]. This sandwich-type immunosensor also included toluidine blue as an electron mediator. The toluidine blue–AuNP–COF labels in association with primary antibody functionalised Au–TiO_2_ nanoparticle doped polypyrrole provided electrochemical responses in a broad concentration range (500 fg mL^−1^–10 ng mL^−1^) and a low LOD (170 fg mL^−1^) [280].

In addition to the aforementioned research, several other studies, listed in Table 5, have been carried out using nanogels, dendrimers, hyperbranched polymers, and COFs for the early diagnosis of cancer, neurological disorders, and diabetes.

### 4.6. Molecularly Imprinted Polymeric Nanoparticles

Molecularly imprinted polymers (MIPs) are artificial materials synthesised by the polymerisation of functional monomers and cross-linking agents in the presence of a template molecule. Following the removal of the template, a cavity is generated which corresponds with the size, shape, and functionality of the template molecule. MIPs thus provide superior physical and mechanical stability in addition to selectivity when compared to their biological equivalents. The synthesis and functionalisation of MIPs is easy and can be sustained in harsh environments. MIP-based nanostructures hold great promise for medical diagnosis applications [294,295,296].

Owing to bioaccumulation from food and dairy products, various microorganisms develop a tolerance towards antibiotics. Antibiotic tolerance hinders the treatment of infectious diseases, and therefore, it is important to know the amount of antibiotics in common biological fluids. Altintas reported a nanoMIP-based SPR sensor for vancomycin detection in milk [297]. In this study, itaconic acid (ITA) was polymerised using trimethylolpropane trimethacrylate and ethylene glycol dimethacrylate as cross-linkers. The nanoMIPs were immobilised on an 11-MUA functionalised Au sensor chip. The sensor displayed high specificity and affinity (dissociation constant: 1.8 × 10^−9^ M) for the antibiotic. A linear range from 10 to 125 ng mL^−1^ was obtained and the LOD was 4.1 ng mL^−1^. Chen et al. developed a surface imprinting strategy for LZM detection [298]. The LZM template was initially immobilised on SiNP and then *N*-(4-vinylbenzyl)iminodiacetic acid (VBIDA) was polymerised in the presence of methylenebisacrylamide, *N*-isopropylacrylamide, and acrylamide. VBIDA chelated with Cu^+2^ ions and recognised LZM due to metal chelate substantivity. The binding kinetics for the enzyme improved significantly due to the surface imprinted nanoparticles. The detection range in this case was 0.1–1.0 mg mL^−1^ and LOD was 0.1 mg mL^−1^. A similar technique was introduced for the photochemical determination of bilirubin, where bilirubin imprinted Fe_3_O_4_–hydroxyapatite(HAP)-PPy nanoparticles were immobilised on a magnetic glassy carbon electrode (MGCE). The biosensor provided a linear response in the 1.0–17 μM range and the detection limit was 0.007 μM. The sensor was highly selective against many interferents and a good affinity was maintained in serum [299].

Yun and co-workers manufactured a piezoelectric sensor for amantadine, a drug used in the treatment of Parkinson’s as well as influenza, using MIP with RGO and AuNPs [300]. Perchlorate (TBAP) was used as a functional monomer in this study. Each step of the sensor fabrication process was optimised. A linear sensor response was recorded between 1.0 × 10^−5^ and 1.0 × 10^−3^ mM with a good sensitivity (LOD: 5.4 × 10^−6^ mM). Denizli’s group developed a MIP based QCM sensor for label-free and highly sensitive determination of synthetic cannabinoids, molecules that mimic the main active component tetrahydrocannabinol of marijuana, in urine [301]. The researchers used 2-methacryloyl-(L)-phenylalanine (MAPA) as the functional monomer. A QCM system could achieve a linear response in the range of 0.0005–1.0 ng mL^−1^ with LODs ranging from 0.2 to 0.45 pg mL^−1^ for four different cannabinoids.

**Figure 13:**
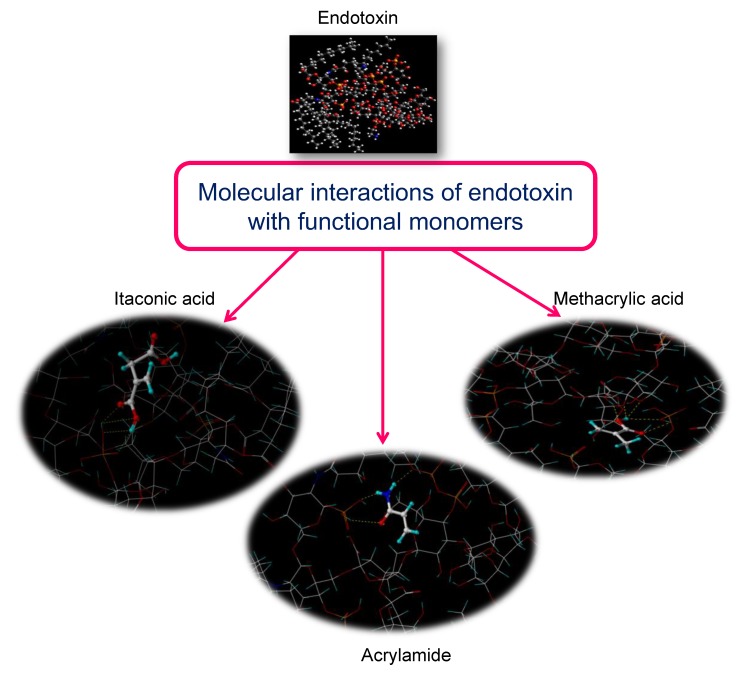
Visualisation of the stoichiometric complex arising from the interaction of endotoxin with three optimum monomers, which were selected using molecular modelling [303].

The application of computational approaches to MIP-based biosensor development holds great promise. Such integrated techniques assist in monomer selection for a particular MIP based on the affinities and binding energies between the monomers, as shown in Figure 13, and the analyte, thus making it possible to narrow down from more than 21 possible monomers and curtail the experimental time and costs [302]. Computational studies can also play a pivotal role in the selection of templates when the analyte is large and structurally complex, such as proteins, viruses, and bacteria. Using this approach, Altintas and co-workers reported a proof-of-concept study suggesting the establishment of epitope libraries to combat the challenges of protein imprinting by simulating the most stabile surface conformation of the neuron specific enolase (NSE) protein in the sensing media [294]. Altintas et al. also reported on ultrasensitive detection of *E. coli* endotoxins using ITA as a functional monomer [303]. The monomer was selected based on in silico monitoring of monomer–endotoxin interaction. The SPR sensor detected the endotoxins in a 15.6–500 ng mL^−1^ range with a limit of 0.44 ± 0.02 ng mL^−1^. Wang and co-workers used AuNP–graphene–MIP(Py) modified GCE to develop an electrochemical levofloxacin sensor [304]. Levofloxacin (LEV) is a common antibiotic used in the treatment of gastroenteritis, pneumonia, and urinary tract infections. The integration of AuNP–graphene in the sensor spurred the electrocatalytic oxidation of LEV [304]. A detection range of 1.0–100 μM was achieved and the LOD was found to be 0.53 μM. The sensor exhibited reproducibility in addition to good specificity and excellent sensitivity. High affinity optical sensing for waterborne viruses was achieved using optical nanoMIP sensors, wherein bacteriophage MS2 was chosen as a template and the functional monomers included *N*-Isopropylacrylamide (NIPAm), acrylic acid (AAc), *N*-tert-butylacrylamide (TBAm), and *N*,*N*′-methylenebis(acrylamide) (BIS) [305]. A state-of-the-art solid phase polymerisation strategy was employed. Virus detection was achieved using an SPR sensor to develop the assay. The high affinity nanosensor achieved an LOD of 5 × 10^6^ pfu mL^−1^ and a detection range of 0.33–27 pM.

### 4.7. Molecular Machines

Molecular machines (MoMa), also known as nanomotors, are miniature devices which are able to move in a liquid phase with the help of an external power source or by self-propulsion. They can be defined as, “an assembly of a discrete number of molecular components designed to perform mechanical-like movements (output) as a consequence of appropriate external stimuli (input)” [306]. Molecular machines are at the cutting edge of scientific development with the 2016 Nobel Prize in chemistry being awarded to Jean-Pierre Sauvage, Sir James Fraser Stoddart, and Bernard (Ben) L. Feringa for their work on “the design and synthesis of molecular machines” [307]. Molecular machines functionalised with biomolecules are expediting the creation of ultramodern and meritorious biosensing systems. They can be conjugated with artificial molecularly imprinted receptors or biological molecules, such as antibodies or ODNs. Owing to their diminutive size and the vortex effect arising from their movement, motion-driven DNA nanomachines have led to ultrasensitive biodetection systems [231,308].

Zhang et al. developed the first resonance Rayleigh scattering (RRS) strategy for uracil-DNA glycosylase (UDG), a base-excision repair enzyme critical to the repair of single-base mutations, by employing a label-free exonuclease III (Exo III) catalysed DNA nanomachine for dual amplification [309]. The nanomachine constituted a double stranded DNA (dsDNA) complex which would dissociate into two single strands that individually hybridise with hairpin probes (HP), triggering dual amplification catalysed by Exo III. The G-quadruplexes formed by the amplification reaction grew into continuous and elongated G-wires in the proximity of Mg^2+^, providing an accentuated RRS intensity. A superior signal-to-noise ratio, along with high sensitivity, was obtained. A low LOD of 1 × 10^−5^ U mL^−1^ and a linear response between 2.0 × 10^−5^ and 0.4 U mL^−1^ was obtained. A qualitative analysis of this biosensing system was also performed successfully in HeLa cells lysate. Another approach using a DNA machine for UDG sensing based on the cleavage of uracil from HPs of the HP–G-quadruplex MoMa resulted in lower sensitivity [310]. In this case, the terminally exposed HPs with G-quadruplex sequences generate a G-quadruplex DNA structure induced by linked bridged hybridisation. The fluorescent intensity was significantly improved using thioflavin T (TfT). The LOD of 7.8 × 10^−3^ U mL^−1^ was thus two orders of magnitude higher than the previous study. 

A similar DNA machine was also reported for the detection on p53, the most common gene mutated in various cancers [311]. Cyclic nucleic acid strand displacement polymerisation (CNDP) was effectuated by p53, triggering the MoMa, which consists of an assistant template (AsT) and HP. The CNDP spurred a catalytic reaction mediated by DNAzyme. This reaction was responsible for the colorimetric detection. The p53 DNA, with the help of polymerase (POx) and nickase (NiOx), drove the MoMa one after the other through displacement cycles involving hybridisation and polymerization, creating a dramatic abundance of G-quadruplexes which mimicked HRP by conjugating with hemin, and were responsible for the 2,2′-azino-bis(3-ethylbenzothiazoline-6-sulfonic acid) (ABTS) oxidation by H_2_O_2_. This resulted in a colour change because of the presence of ABTS^•+^ oxidation product. The system provided a wide linear response range from 10^−14^ to 10^−7^ M with a limit of 10 fM.

Hou’s group developed a sensitive, highly specific, and reusable aptasensor to obtain a sandwich HαT immunoassay using a DNA machine comprising two distinct biotinylated thrombin aptamers (TBA), each immobilised on a streptavidin functionalised MNP as resonance light scattering (RLS) probes [312]. The novel sensor could offer a detection in a wide concentration range (60 pM–6.0 nM). Cao et al. developed a catalyst-fuelled molecular machine capable of detecting the CD63 exosome [313]. Primarily, CD63 antibodies were immobilised on immune magnetic beads (IMB). Once the exosome bound to the anti-CD63-IMB probe, it was recognised by a CD63 aptamer, which launched the MoMa. The MoMa was propelled due to a cascade toehold-mediated strand displacement reaction (CTMSDR). The products from CTMSDR were then transferred onto a gold electrode functionalised with a dibenzocyclooctyne (DBCO). The LOD of the biosensing system was 1.72 × 10^4^ particles mL^−1^. The “proof-of-concept” method displayed enormous potential for clinical applications due to its highly specific sensing in serum samples. Zhao and co-workers developed a similar 3D DNA machine induced by immunorecognition for the detection of serum cystatin C (CstC), a biomarker of nephrological and cardio-vascular diseases, relying on toehold-mediated strand displacement reaction (TMSDR) [314]. They coated a GCE with GO functionalised with a monolayer of rubrene (mRub) and platinum nanoparticles. Further modification with βCD and BSA was performed to obtain a high signal amplification. Fe_3_O_4_ functionalised AuNPs were used as a substrate for the construction of a 3D DNA machine composed of thiolated substrate DNA (TSDNA), ferrocene-labeled assistant strands (FcAS), and ferrocene-labeled blocker strand (FcBS). A sandwich immunocomplex formed by capture antibody walking DNA (cAbWD) displaced the FcBS to allow the fuel strand (FuSt) to hybridise with TSDNA. The ensuing TMSDR provided energy to the sandwich immunocomplex resulting in an enormous of release of FcAS and FcBS. These strands were magnetically collected and conjugated with the βCD of the electroluminescent system, quenching the signal in proportion with the CstC concentration. The ultrasensitive sensor exhibited a linear response from 1.0 fg mL^−1^ to 10 ng mL^−1^ with an LOD of 0.38 fg mL^−1^.

Bagheryan’s group prepared a Gquadruplex machine for biosensing meso-5,10,15,20-tetrakis-(*N*-methyl-4-pyridyl)porphine (TMPyP4), an anticancer drug [315]. The nanomachine was prepared on an SPCE using a mesoporous structure of (4-(*N*-methyl-*N*-(carboxypropyl)amino)-phenyl-4′-(*N*,*N*-dibutylamino)phenylsquaraine (SBA)-enriched AuNPs with *N*-propylpipyrazine-*N*-(2-mercaptopropane-1-one) (NNPNSH). Because of these modifications, the AuNPs were able to bond with thiolated Gquadruplex DNA (SHG_4_DNA) in the presence of TMPyP4. They obtained highly reproducible signals with DPV for TMPyP4 concentrations between 0.1 and 10 μM. Zhen et al. have recently reported an electrochemiluminescent sensor for the detection of folate receptor (FoR), a biomarker which is overexpressed in several types of cancers, using a folic acid (FoA) labelled DNA nanomachine [316]. In the absence of the target biomarker, the strand displacement amplification (SDA) of the nanomachine takes place with the help of NiEnOx and POx, which release peroxide-mimicking DNAzymes responsible for chemiluminescence. However, when FoR was present, the NiEnOx based SDA was prevented. The different signals in the former and latter cases were responsible for a high sensitivity and a low LOD of 1 pM with a quasilinear logarithmic response for concentrations ranging between 5 pM and 50 nM.

**Figure 14:**
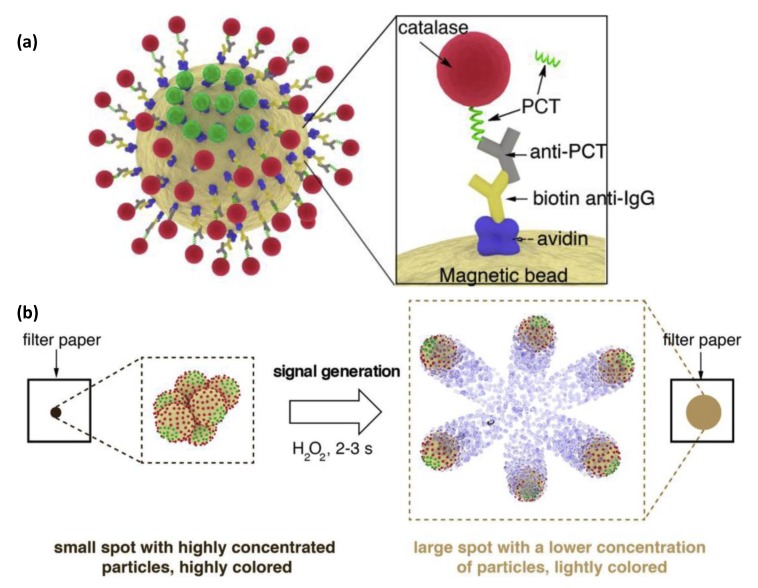
(**a**) Molecular machine with a Janus coating responsible for biorecognition, and iron oxide core providing colorimtric and magnetic properties (**b**) Signal generation mechanism: after spotting the particles on a piece of filter paper H_2_O_2_ is added; the catalase enzymes generate bubbles that propel the particles and disperse the color within seconds. The subsequent change in pixel intensity is read in real time with a mobile phone app [317].

Russel et al. developed an interesting approach to detect the sepsis biomarker procalcitonin (PCT) [317]. They used self-propelled Janus nanoparticles for the colorimetric determination of particle motion. The surface of Janus nanoparticles displays two or more different physical properties on distinct regions of the same particle. In this particular biosensor, avidin (AVN) functionalised Fe_3_O_4_ MNPs were first coated asymmetrically with biotinylated antibodies and then the remaining free sites were coated with biotinylated BSA. This hybrid surface, shown in Figure 14a,b, encouraged self-propulsion when a sandwich assay with the help of catalase (CTOx) was formed. The state-of-the-art biosensor could detect as little as 0.4 ng mL^−1^ PCT.

### 4.8. Polymer Nanocomposites

Polymer nanocomposites (PNCs) possess a variety of morphologies in addition to smart responsibilities and facile synthesis techniques. These unconventional qualities are responsible for the biocompatibility, environmental stability, superior electronic behaviour, and cost-effective nature of PNCs. PNCs are compatible with a variety of biomolecules, such as ODN, aptamers, proteins, and enzymes to enable biorecognition. Their large surface area and fast electron transfer rate in combination with the aforementioned characteristics make them ideal candidates for signal transduction in biosensing systems [230].

Robby and Park fabricated a colorimetric bacterial biosensor employing a nanocomposite of polymer dots (PDs) intercalated into montmorillonite (MMT) [318]. The PDs were made of carbonised boronic acid conjugated catechol–conjugated polyethylene glycol–grafted poly(dimethylamino)ethyl methacrylate and contained βCD. Fe_3_O_4_ and CsWO_3_ were also immobilised on the nanocomposite as recyclable metallic nanoparticles. Fluoerescence quenching of the p-nitrophenyl phosphate (NPP) subtrate was achieved with alkalinephosphatase (ALPOx). The fluorescence-based biosensor provided a broad detection range for *E. coli* and *S. aureus* between 10^1^ and 10^8^ CFU mL^−1^, with respective LODs of 5.25 cfu mL^−1^ and 5.45 cfu mL^−1^. Another nanocomposite combining polymer with clay was reported by Emre et al. for glucose biosensing [319]. The PNC consisted of poly(methylmethacrylate) (PMMA), 4-(2,3-dihydrothieno [3,4-b][1,4]dioxin-5-yl)-7-(2,3-dihydrothieno[3,4-b][1,4]dioxin-7-yl)-2-benzyl-1H-benzo[d] imidazole polymer (PBIPE), and the synthetic clay laponite (Lap). PBIPE is a conductive polymer with aromatic units that enable the hosting of GOx and PMMA-Lap with high efficacy. It acted as an excellent electron carrier and encouraged charge transfer. The nanocomposite enhanced the enzyme immobilisation and also contributed to signal amplification, leading to a detection range from 51 and a limit of 37.6 μM. Another study also reported a glucose-based biosensor, using a nanocomposite of poly(brilliant cresyl blue) (pBCB) and MWCNT [320]. The use of ethaline deep eutectic solvent for the electro polymerisation of BCB significantly improved the sensing characteristics. The enzymatic sensor displayed an LOD of 2.9 μM. The same sensor could also be employed for CAT detection when GOx was substituted with TSA. The LOD for CAT detection was found to be 3.9 μM.

Nguyen and co-workers reported a facile strategy to fabricate a glutamate sensor using a nanocomposite ink consisting of the ICP poly (3,4-ethylenedioxythiophene) polystyrene sulfonate (PEDOT-PSS) with MWCNTs, PtNPs and silicone rubber (SiRb) [321]. Finally, glutamate oxidase (GMTOx) was immobilised on nanocomposite. The ink was ultimately written on a PDMS substrate. The sensor was found to be highly selective, with linear responses between 10 and 600 μM and an LOD of 0.2 μM. The biosensing system was also employed to characterise the glutamate release from the spinal cord of a rat. It showed tremendous potential for application in implantable glutamate biosensors for patients with neurotrauma. Divya et al. reported a polyindole(Pin)–zerovalent silver nanocomposite for the detection of DA at immiscible liquid/liquid interfaces with nanomolar sensing abilities [322]. Fluorescence measurements provided an extremely wide linear detection response from 40 nM to 12 μM.

PNCs are also frequently used for monitoring pharmaceutical drugs. Phukon et al. reported a polyhydroxyalkanoate (PHOA)–gold nanocomposite for artemisinin determination [323]. Artemisinin is a potent antimalarial drug. PHA was extracted from natural sources and the nanocomposite film was mounted on an ITO electrode prior to HRP immobilisation. No cross-linker was required in the fabrication of the biosensor. Linearity was observed from 10.0 to 60.0 ng mL^−1^ in spiked human serum with an LOD of 3.6 ng mL^−1^. Yupintharakun’s group developed an optosensor for the detection of ciprofloxacin, an antibiotic commonly used in the treatment of various ailments of the skin and kidneys, by employing a nanocomposite of CMWCNT, CdTe quantum dots, and molecularly imprinted APTES polymer [324]. The sensor showed high binding affinity and specificity, and exceptional sensitivity wherein the fluorescence intensity reduced in a linear fashion from 0.10 to 100.0 μg L^−1^ and the detection limit was found to be 66 ng L^−1^.

Soni et al. used PAni–MoS_2_ nanoflower composites for the detection of chronic myelogenous leukemia (CML), a leukemia biomarker [325]. The bioelectrode used to develop the impedimetric sensor consisted of ITO and plasmid DNA (pDNA) withal the nanoflowers. EIS provided a very wide detection range of 10 aM to 1 μM and the LOD was 3 aM. The genosensor was also tested with a variety of DNA sequences and real samples to exhibit its potential for healthcare diagnostics.

Further research works, relying on nanoMIPs, nanomotors and polymeric nanocomposite-based sensors, are listed in Table 6.

## 5. Future Prospects

Nanomaterials, by a combined virtue of their small size and exceptionally high surface area, exhibit interesting electromagnetic, optical, and piezoelectric properties, which hold immense potential for exploitation in the disciplines of medical diagnostics, bioimaging, and healthcare diagnostics. In addition, nanomaterials possess excellent affinity for biomolecules, facilitating the immobilisation of antibodies, enzymes, nucleic acids, proteins, and many other clinically relevant substances, opening up possibilities to develop a wide variety of sensing platforms, such as aptasensors, immunosensors, enzymatic sensors, sandwich assays, and many others. Recent advancements in biosensing platforms have employed various novel forms of nanomaterials, ranging from monomolecular nanomotors to relatively larger nanocages. The rapid, cost-effective, and facile operational procedures offered by nanomaterial-based biosensors are expected to overhaul conventional expensive sensing systems in the near future.

## Figures and Tables

**Table 1:** Carbon allotrope-based electrochemical biosensors.

Sensor Platform/Label	Analyte	Detection Mechanism	Detection Range	LOD	Reference
GCE–Ph–GO–ERC_60_	HVA	CV, SWV, EIS	0.1–7.2 μM	0.03 μM	[60]
PGE–vinyllic C_60_–polyTAT	PQ	DPASV, CV	2.7–848.5 nM	800 pM	[61]
GCE–C_60_–MWCNT	AC LD	CV, CA, DPV	0.1–1.5 mM 0.5–2000 μM	0.43 μM 35 nM	[62]
CPE–C_60_–MWCNT–CuNPs	PT	SWV	4.0–400 nM	73 pM	[63]
GCE–C_60_–GO	DA	CV, DPV	0.02–73.5 μM	8 nM	[64]
SPCE–AC_60_–PdNPs	DA	DPV	0.35–133.35 μM	56 nM	[65]
Graphite–SWCNT–TTF–ADH	Glycerol	CV	0.05–1.0 mM	18 µM	[66]
GCE–Ni(TPA)–SWCNT	Glucose	CV, CA	20 μM–4.4 mM	4.6 μM	[67]
GCE–CuAu–CNTs–CSs–anti-CEA	CEA	LSV	0.025–25 ng mL^−1^	0.5 pg nL^−1^	[68]
GCE–3D graphene@Au NCs–MWCNTs–NH_2_–BCERAb_1_/ssDNA–BCERAb_2_	MCF-7	SWV, EIS, DPV	1.0 × 10^2^–1.0 × 10^6^ cells mL^−1^	80 cells mL^−1^	[69]
SWCNT–SPE–PNA/biotin PNA	DNA	DPV	0.25–1.75 nM	71 pM	[29]
GCE–CuFe_2_O_4_/RGO–AuNPs	Cys	CV, EIS	0.05–2 mM	0.383 µM	[70]
Au–CDs–ZrHf@MOF–aptamer	HER2 MCF-7	EIS, DPV	0.001–10 ng mL^–1^ 100–10,000 cell mL^–1^	19 fg mL^–1^ 23 cell mL^–1^	[71]
GCE–CD–AuNPs–DNA	BCR-ABL1	DPV	10 pM–100 μM	1.5 pM	[72]
GCE–CD–AuNPs–CEA aptamer	CEA		1 × 10^−9^–1 mg mL^−1^	0.26 pg mL^−1^	
Au–ND–LOx	Lactate	CV, DPV	0.05–0.7 mM	15 µM	
GCE–ND–DHP	COD	SWV	0.299–10.8 μM	54.5 nM	[73]
GCE–ND–PoSt–Tyr	CAT	DPV	5–740 μM	0.39 μM	[43]

Abbreviations: 3D: 3 dimensional; AC: Acetamenophen; AC60: Activated fullerene; ADH: Alcohol Dehydrogenase; anti-: Antibody of; BCR-ABL1: Breakpoint cluster region protein Abelson murine leukaemia homolog 1; BCERAb: Breast Cancer Estrogen Response Antibody; C60: Fullerene; CA: Chronoamperometry; CAT: Catechol; CC: Chronocoulometry; CDs: Carbon dots; CEA: Carcinoembryogenic antigen; CNT: Carbon nanotubes; COD: Codeine; CPE: Carbon paste electrode; CSs: Microcarbon spheres; CV: Cyclic voltammetry; Cys: Cysteine; DA: Dopamine; DHP: Dihexyl phosphate; DNA: Deoxyribonucleic acid; DPASV: Differential pulse anodic stripping voltammetry; DPV: Differential pulse voltammetry; ERC60: Electrochemically reduced fullerene; EIS: Electrochemical impedance spectroscopy; GCE: Glassy carbon electrode; GO: Graphene oxide; HVA: Homovanilic acid; HER2: Human epidermal growth factor receptor 2; LD: Levadopa; LOx: Lactate oxidase; LSV: Linear sweep voltammetry; MCF-7: Michigan cancer foundation-7 cell line; MOF: Metal organic framework; MWCNT: Multi-walled carbon nanotubes; NP: Nanoparticle; NCs: Nanocubes; ND: Nanodiamond; PGE: Pencil graphite electrode; Ph: Phenyl modification; PNA: Peptide nucleic acid; polyTAT: poly(2,4,6-trisacrylamido-1,3,5-triazine); PQ: Primaquone; PT: Paracetamol; PoSt: Potato starch; RGO: Reduced graphene oxide; SPCE: Screen printed carbon electrode; SPE: Screen printed electrode; ssDNA: Single strand DNA; SWCNT: Single walled carbon nanotubes; SWV: Square wave voltammetry; TPA: Terephthalic acid; TTF: Tetrathiofulvalene; Tyr: Tyrinose.

**Table 2:** Carbon allotrope-based biomedical diagnostics using optical, piezoelectric, and other types of sensing techniques.

Sensor Platform/Label	Analyte	Detection Mechanism	Detection Range	LOD	Reference
LFS–Str/CMWCNT–DNA	Hg^2+^	VE, IJS	0.05–1 ppb	0.05 ppb	[129]
LFS–MWCNTs–DNA/biotinylated DNA	DNA	VE, IJS	0.1–20 nM	0.004 nM	[130]
Gelatine stabilised RGO–AuNP	Cys	UV-vis, FS	0.51–0.4 μM	0.51 nM	[99]
Eu–GQD	Cu^+2^ Cys	UV-vis, FS	0.1–10 μM 0.5–50 μM	0.056 μM 0.31 μM	[100]
CD–PAMAM–RGDS	Cu^+2^	UV-vis, FS	0.01–2 mM	0.01 μM	[131]
CD–anti-Desmin	Desmin	PL	0.714–4.286 ng mL^−1^	<1 ng mL^−1^	[41]
IDE–SWCNT–pleurocidin	*E. coli* *E. faecalis* *C. albicans*	MSPQC	10–10^3^ cfu mL^−1^ 100–10^3^ cfu mL^−1^ 100–10^3^ cfu mL^−1^	10 cfu mL^−1^ 100 cfu mL^−1^ 100 cfu mL^−1^	[82]
IDE–SWCNT–Anti-GAS aptamer	GAS	SPQC	3 × 10^2^–3 × 10^6^ cfu mL^−1^	12 cfu mL^−1^	[84]
Fe_3_O_4_–ND–GO	SLC	HPLC-DAD	5.00–250.00 ng mL^−1^	1.49 ng mL^−1^	[132]

Abbreviations: anti-: Antibody of; *C. albicans*: *Candida albicans*; CD: Carbon dot; CMWCNT: Carboxylated multiwalled carbon nanotubes; Cys: Cysteine; DNA: Deoxyribonucleic acid; *E. coli*: *Escherichia coli*; *E. faecalis*: *Enterococcus faecalis*; GAS: Group A *Streptococcus*; GO: Graphene oxide; GQD: Graphene quantum dots; HPLC-DAD: High performance liquid chromatography–diode array detector; IDE: Interdigitated electrode; IJS: ImageJ Software; LFS: Lateral flow strip; MSPQC: Multichannel series piezoelectric quartz crystal; MWCNT: Multiwalled carbon nanotube; ND: Nanodiamond; NP: Nanoparticle; PAMAM: Poly(amidoamine); PL: Photoluminescence; ppb: parts per billion; RGDS: Arginine-glycine-aspartic acid-serine; RGO: Reduce graphene oxide; SPQC: Series piezoelectric quartz crystal; Str: Streptavidin; SWCNT: Single walled carbon nanotubes; SLC: Seldinafil citrate; UV-vis: Ultraviolet-visible spectroscopy; VE: Visual evaluation.

**Table 3:** Inorganic nanomaterial-based optical, piezoelectric, and other biosensors.

Sensor Platform/Label	Analyte	Detection Mechanism	Detection Range	LOD	Reference
Au-carboxylMNP	PSA	SPR	10^−4^–1 μg mL^−1^	100 pg mL^−1^	[166]
Au-MUC-1/MNP-FA	MCF-7	SPR	5 × 10^2^–10^4^ cells mL^−1^	500 cells mL^−1^	[167]
AgNP-DTNB-McAb-BSA/Fe_3_O_4_-APTES-PcAb-BSA	MMP-9	SERS	1 pg mL^−1^–100 ng mL^−1^	1 pg mL^−1^	[168]
Au-MPA-pAb-EA/carboxylMNP-pAb-EA	*S. enteritidis*	SPR	1.4 × 10^1^–1.4 × 10^9^ cfu mL^−1^	14 cfu mL^−1^	[169]
CdS NanoCrys-thiol anti-p53-BSA/biotin anti-p53-AuNP-GO	p53	ECL	20–1000 fg mL^−1^	4 fg mL^−1^	[170]
GCE-chit-Au_shell_-GOx_core_-GOx	Glucose	ECL	1.0 μM–4.3 mM	0.3 μM	[138]
MEA-Au-film-GO-anti-IgM/AuBPs	IgM	LSPR	0.03–32 μg mL^−1^	0.03 μg mL^−1^	[141]
probeDNA/Exo-III-Ag NPC	Coralyne	FS	5–1000 nM	1.83 nM	[171]
AuNP-HyA-PTA/AuAg NCPs	HAase	FS	0.5–37.5 U mL^−1^	0.3 U mL^−1^	[172]
Ag_shell_-AuNR_core_	p-AP	VE, SPR	1–70 μM	0.64 μM	[173]
carboxylFe_3_O_4_-mAb	Ricin	TMR	1 × 10^−3^–200 μg mL^−1^	1 ng mL^−1^	[174]
APTES@silica Fe_3_O_4_-aptamer	AFM1	HPLC	0.3–50 ngL^−1^	0.2 ngL^−1^	[175]
Fe_3_O_4_NPC-Str-biotin dAb/cAb	*Salmonella*	NMR	10^5^–10^7^ cfu mL^−1^	10^5^ cfu mL^−1^	[176]
Ab_1_/Ab_2_-Glut-AuNPs	CD-10	QCM	10–100 pM	2.4 pM	[139]

Abbreviations: Ab: Antibody; AFM1: Aflatoxin M1; anti-: Antibody of; APTES: (3-Aminopropyl)triethoxysilane; BPs: Bipyramidal nanoparticles; BSA: Bovine serum albumin; cAb: Capture antibody; CD-10: Cluster of differentiation 10; chit: Chitosan; dAb: Detection antibody; DTNB: 5,5’-dithiobis-(2-nitrobenzoic acid); EA: Ethanolamine; ECL: Electrochemiluminescence; FA: Folic acid; FS: Fluoerescence spectroscopy; GCE: Glassy carbon electrode; GO: Graphene oxide; GOx: Glucose oxidase; Glut: L-Glutathione; HAase: Hyaluronidase; HPLC: High performance liquid chromatography; HyA: Hyaluronic acid; IgM: Immunoglobulin M; LSPR: Localised surface plasmon resonance; mAb: Monoclonal antibodies; McAb: Matrix Metalloproteinases 9 detection antibody; MCF-7: Michigan cancer foundation-7 cell line; MEA: 2-mercaptoethylamine; MMP-9: Matrix Metalloproteinases 9; MPA: 3-Mercaptopropionic acid; MNP: Magnetic nanoparticles; MUC-1: Human mucin-1; NanoCrys: Nanocrystal; NCPs: nanocluster particles; NIR: Near infrared; NMR: Nuclear magnetic resonance; NP: Nanoparticle; NPC: Nanoparticle clusters; NR: nanorods; p53: Phosphoprotein 53; pAb: Polyclonal antibodies; p-AP: p-aminophenol; PcAb: Matrix Metalloproteinases 9 capture antibody; PSA: Prostate Specific Antigen; PTA: Protemine; QCM: Quartz crystal microbalance; *S. enteritidis*: *Salmonella enteritidis*; SERS: Surface enhanced Raman spectroscopy; SPR: Surface plasmon resonance; Str: Streptavidin; thiol: Thiolated; TMR: Tunnelling magnetoresistance; UV-vis: Ultraviolet-visible spectroscopy; VE: Visual Evaluation.

**Table 4:** Inorganic nanomaterial-based electrochemical biosensors.

Sensor Platform/Label	Analyte	Detection Mechanism	Detection Range	LOD	Reference
carboxylMNP-Str-mAb/AuNP-urease-pAb	*L. monocytogenes*	EIS	3 × 10^2^–3 × 10^4^ cfu mL^−1^	30 cfu mL^−1^	[204]
Au-IDE-thiourea-carboxylAuNP-mAb	CEA hEGFR CA 15-3	Capacitance	20–1000 pg mL^−1^ 20–1000 pg mL^−1^ 10–100 U mL^−1^	5 pg mL^−1^ 5 pg mL^−1^ 1 U mL^−1^	[188]
NH_2_-AgNP-CNT-GO-aptamer	DA	DPV	3–110 nM	700 ± 19.23 pM	[205]
Au-MCH-peptide:GO-AgNP	PSA	EIS, CV, LSV	0.005–20 ng mL^−1^	0.33 pg mL^−1^	[206]
GCE/Cu_2_O NShs/Nafion	Glucose	Amperometry	1.25–37.5 μM	0.4 μM	[207]
GCE-hAuRuNShs	AA	LSV	5 μM–2 mM	2.2 μM	[208]
GCE-CMG-Au NCgs	Hydrazine	Amperometry	6–1600 μM	0.5 μM	[209]
GCE-SGN-PdPt NCgs	H_2_O_2_	Amperometry	1–300 μM	0.3 μM	[210]
GCE-choline-Au NCgs-MWCNT	GMP	CV, DPV	0.3–600 μM	0.1 μM	[211]
Si NW-anti-8-OHdG	8-OHdG	i-v char	3.5–141 nM	3.5 nM	[212]
Si-ZnO NWA-Au_film_-MPA-LZM	Ig	i-v char	5 × 10^2^–1 μg mL^−1^	102.76 ng mL^−1^	[213]
PPCE-ZnO NW-GOx	Glucose	CA	0.1–3.6 mM	46 ± 31 µM	[214]

Abbreviations: 8OHDG: 8-hydroxydeoxyguanosine; AA: Ascorbic acid; anti-: Antibody of; CA: Chronoamperometry; CA 15-3: Cancer antigen 15-3; CEA: Carcinoembryogenic antigen; CMG: Chemically modified graphene; CNT: Carbon nanotube; CV: Cyclic voltammetry; DA: Dopamine; DPV: Differential pulse voltammetry; EIS: Electrochemical impedance spectroscopy; GCE: Glassy carbon electrode; GO: Graphene oxide; GOx: Glucose oxidase; GMP: guanosine-5′-monophosphate; hAuRuNShs: Hollow gold ruthenium nanoshells; hEGFR: Human epidermal growth factor receptor; IDE: Interdigitated electrode; Ig: Immunoglobulin; i-v char: i-v characterisation; *L. monocytogenes*: *Listeria monocytogenes*; LSV: Linear sweep voltammetry; LZM: Lysozyme; mAb: Monoclonal antibody; MCH: Mercaptohexanol; MNP: Magnetic nanoparticles; MPA: 3-Mercaptopropionic acid; MWCNT: Multiwalled carbon nanotubes; NCgs: Nanocages; NP: Nanoparticles; NShs: Nanoshells; NW: Nanowire; NWA: Nanowire array; pAb: Polyclonal antibody; PSA: Prostate specific antigen; SGN: SnO_2_-graphene nanosheets; Str: Streptavidin.

**Table 5:** Biosensors based on dendrimers, nanostructured hydrogels, and hyperbranched polymers.

Sensor Platform/Label	Analyte	Detection Mechanism	Detection Range	LOD	Reference
DDCE-(PAMAM)-CATM-BSA/chit-AgI-anti-IL-6	IL-6	PEC	10 ag mL^−1^–90 pg mL^−1^	3.3 fg mL^−1^	[281]
DDCE-(PAMAM)-CATM-BSA/g-C_3_N_4_-anti-PSA	PSA	PEC	1 ag mL^−1^–90 pg mL^−1^	33 ag mL^−1^	[281]
Au-NHg(poly(HEMA-EGDM-APBA))	Glucose	FS (LSPR)	1–50 mM	50 μM	[282]
ITO-NHg(FMOC-Lys-FMOC-Pha)-Cyt C-FTIC	H_2_O_2_	Amperometry	3 × 10^−7^–8 × 10^−4^ M	50 nM	[283]
GCE-NHg(Frc-Pha)-GOx-chit	Glucose	Amperometry	0.1–20 mM	50 μM	[284]
GCE-NHg(PAni)-PtNP-UOx	UA	Amperometry	0.07–1 mM	1 μM	[285]
GCE-NHg(PAni)-PtNP-ChsOx-ChEt	Cholesterol	Amperometry	0.3–9 mM	0.3 mM	[285]
GCE-NHg(PAni)-PtNP-LIP-GK-GPO	Triglyceride	Amperometry	0.2–5 mM	0.2 mM	[285]
Au-NHg(NAS-BSA-GOx)	Glucose	CV	0.0–0.6 mM	1.116 × 10^−2^ M	[286]
NHg(PPy-AuNP)-anti-CEA-BSA	CEA	DPV	1.0 × 10^−6^–200 ng mL^−1^	160 ag mL^−1^	[287]
Pt-HBP(PDAMS)-PtNP	NADH	CA	87–2500 μM	4.78 μM	[288]
Pt-HBP(PMDUS)-PtNP	NADH	CA	0–2100 μM	6.18 μM	[288]
HBP(PAMAM-NH_2_) AgNP_shell_-chit_core_	NH_3_	UV-vis	10–50 ppm	8 ppm	[289]
2D-COF (Zr-amide-por)-MIP(*o*-PD)-GCE	Tetracycline	ECL	5–60 pM	2.3 pM	[290]
COF (porphyrin)-CRP aptamer	CRP	PEC	0.5–100 ng mL^−1^	100 pg mL^−1^	[291]
COF(TpPa)-MIP(MAA-AM)-CD	Tryptamine	Fluorescence	0.025–0.4 mg kg^−1^	7.0 μg kg^−1^	[292]
COF(TBAPy-MA)-COOH/AgNCgs_shell_AuNP_core_	miRNA 155	DPV	10 fM–1 nM	6.7 fM	[293]
COF(TBAPy-MA)-COOH/Cu_2_O_shell_AuNP_core_	miRNA 122	DPV	10 fM–1 nM	1.5 fM	[293]

Abbreviations: AM: Acrylamide; anti-: Antibody of; APBA: Aminophenylboronic acid; BSA: Bovine serum albumin; CA: Chronoamperometry; CATM: Cube anatase TiO_2_ mesocrystals; CD: Carbon dots; CEA: Carcinoembryogenic antigen; ChEt: Cholesterol esterase; chit: Chitosan; ChsOx: Cholesterol oxidase; COF: Covalently organic framework; CRP: C-reactive protein; CV: Cyclic voltammetry; Cyt C: Cytrochrome C; DDCE: Dual disk glassy carbon electrode; DPV: Differential pulse voltammetry; ECL: Electrochemiluminescence; EGDM: Ethylene glycol dimethacrylate; FMOC: Fluorenylmethyloxycarbonyl protecting group; Frc: Ferrocene; FS: Fluorescence spectroscopy; FTIC: Fluorescein Isothioscyanate; g-C_3_N_4_: Graphitic carbon nitride; GCE: Glassy carbon electrode; GK: Glycerol kinase; GOx: Glucose oxidase; GPO: glycerol-3-phosphate oxidase; HBP: Hyperbranched polymer; 2-hydroxyethyl methacrylate; IL-6: Human interleukin 6; ITO: Indium tin oxide; LIP: Lipase; LSPR: Localised surface plasmon resonance; Lys: Lysine; MAA: Methacrylic acid; MIP: Molecularly imprinted polymer; NADH: dihydronicotinamide adenine dinucleotide; NAS: *N*-Succinimidyl Acrylate; NHg: Nanostructured hydrogels; NP: Nanoparticles; *o*-PD: Ortho-phenylenediamine; Pa: *p*-Phenylenediamine; PAMAM: Polyamidoamine; PAni: Polyaniline; PDAMS: poly(diallylmethylsilane); PEC: Photoelectrochemical analysis; Pha: Phenylalanine; PMDUS: poly(methyldiundecenylsilane); por: Porphyrin; ppm: Parts per million; PPy: Polypyrrole; PSA: Prostate specific antigen; Tp: 1,3,5-triformylphloroglucinol; UA: Uric acid; UOx: Uricase; UV-vis: UV-visible spectroscopy.

**Table 6:** Biosensors based on MIPs, molecular machines, and polymer nanocomposites.

Sensor Platform/Label	Analyte	Detection Mechanism	Detection Range	LOD	Reference
Au-MIP(Scp)	Trf	SWV, SPR	0.1–1 μM	0.1 μM	[326]
Au-MIP(HEMA-MAAsp)	LOV	QCM	0.10–1.25 nM	0.030 nM	[327]
Au-MIP(HEMA-Trp)	Bilirubin	QCM	1–50.0 μg mL^−1^	0.45 μg mL^−1^	[328]
ISE-MIP(Acr)	Cocaine	Potentiometry	1 nM–1 mM	1 nM	[329]
SPCE-MWCNT-MIP(4-ABA)	Naloxone	DPV	0.25–10.0 μM	0.20 μM	[330]
GCE-MWCNT-MIP(Acr)	HIV-p24	CV, DPV	0.1–2000 pg mL^−1^	0.083 pg mL^−1^	[331]
Au-DFC-MIP(AM-BA)	PSA	SPR	81–650 nM	81 nM	[332]
Au-MIP(HEMA-MCME)-Fe^3+^	UA	SPR	0.5–40 mgL^−1^	0.247 mgL^−1^	[333]
MoMa(HP-PclPT)-POx-NiEnOx-hemin-ABTS-H_2_O_2_	K-ras	Colorimetry	0.01–150 nM	10 pM	[334]
MoMA(TrgT-CDNAMB-gDNA)-POx-NiEnOx-AmpT	miRNA	Colorimetry	10 aM–1.0 nM	5 aM	[335]
MoMa(scDNAMB)-POx-RsEnOx	p53	FS	0.1 pM–200 nM	0.1 pM	[336]
Au-MoMa(DNA tetrahedron)-NiEnOx-ThAxP	DNA	EIS	10 fM–10 pM	3.7 fM	[337]
MoMa(CMB-PP)-POx-NiEnOx	K-ras	FS	50 pM–10 nM	50 pM	[338]
Au-MoMa (Exo-III-HADT-WD-LoPr)	Ampicillin	DPV	1 pM–10 nM	0.76 pM	[339]
GCE-MoMa(GDHOx-NAD^+^-[Ru(bpy)_3_]^2+^)	Glucose	ECL	5–40 mM	5 mM	[340]
MoMa(DNA[AsPr-SP-CP]-FuSt-Dabcyl-FAM)	Hg^2+^	FS	0.1–100 nM	65 pM	[341]
MoMa(G-quadruplex-NMM)	AFM1	FS	0.01–2.0 ng mL^−1^	0.01 ng mL^−1^	[342]
PNC(PPy_shell_-CeO_2_NR_core_)-ssDNA	*Salmonella*	CV, EIS	10–400 pM	84 pM	[343]
PNC(Au-PDA-CuInZnS QD)	p53	ECL (SPR)	0.1–15 nM	0.03 nM	[344]
FTO-PNC(PAni-Au-Ag-GFN)	AA	CA	1 pM–10 mM	<1 pM	[345]
PNC(Phc-PSty)	CA-125	FS	0.01–127 U mL^−1^	10^−4^ U mL^−1^	[346]

Abbreviations: [Ru(bpy)_3_]^2+^: Tris(bipyridine)ruthenium(II); 4-ABA: 4-aminobenzoic acid; AA: Ascorbic acid; ABTS: 2,2′-azino-bis(3-ethylbenzothiazoline-6-sulfonic acid); Acr: Acrylamide; AFM1: Aflatoxin M1; AM-BA: 3-acrylamidophenyl)boronic acid; AmpT: Amplification Template; AsPr: Assistant Probe; CA: Chronoamperometry; CA-125: Cancer antigen 125; CDNAMD: C-rich DNA modified molecular beacon; CMB-PP: Cyclised Molecular Beacon-Embedded Padlock Probe; Cp: Capture probe; CV: Cyclic voltammetry; Dabcyl: [4-((4-(dimethylamino)phenyl)azo)benzoic acid]; DFC: Acrylamide-alkyne cysteine derivative; DNA: Deoxyribonucleic acid; DPV: Differential pulse voltammetry; ECL: Electrochemiluminescence; EIS: Electrochemical impedance spectroscopy; Exo-III: Exonuclease-III; FAM: 6-carboxyfluorescein; FS: Fluorescence spectroscopy; FTO: Fluorine doped tin oxide electrode; FuSt: Fuel strand; GCE: Glassy carbon electrode; GDHOx: Glucose dehydrogenase; gDNA: Genomic DNA; GFN: graphene family nanomaterials; HADT: Hemin aptamer containing DNA track; HEMA: 2-hydroxyethyl methacrylate; HIV-p24: Human immunodefiiency virus p24 capsid protein; HP: Hairpin probes; ISE: Ion selective electrode; K-ras: Kirsten Rat Sarcoma oncogene; LoPr: Locking probe; LOV: Lovastatin; MAAsp: Methacryloylamidoaspartic acid; MCME: methacryloyl-l-cysteine methyl ester); MIP: Molecularly imprinted polymer; miRNA: microribonucleic acid; MoMa: Molecular machine; MWCNT: Multiwalled carbon nanotube; NiEnOx: Nicking Endonuclease; NAD^+^: Nicotinamide adenine dinucleotide; NMM: *N*-methyl-mesoporphyrin; NR: Nanorods; p53: Phosphoprotein 53; PAni; Polyaniline; PclPT: primer-contained linear polymerization template; PDA: Polydopamine; PNC: Polymer nanocomposite; POx: Polymerase; PPy: Polypyrrole; PSty: Polystyrene; QCM: Quartz crystal microbalance; RsEnOx: Restriction endonuclease; scDNAMB: Short Cleaved DNA molecular beacon; Scp: Scopoletin; SP: Signalling probe; SPCE: Screen printed carbon electrode; SPR: Surface plasmon resoanance; ssDNA: single strand DNA; SWV: Square wave voltammetry; ThAxP: Thiolated Auxiliary Probe; Trf: Transferrin; Trp: L-tryptophan methyl ester; TrgT: Trigger Template; UA: Uric acid; WD: Walker DNA.
